# Algal bioactive compounds for next-generation biopesticides: a multi-target resource for sustainable pest management

**DOI:** 10.1007/s11356-026-38228-3

**Published:** 2026-09-16

**Authors:** Adegoke Isiaka Adetunji, Jeff David Ojwach, Rahul Kumar Goswami, Ismail Rawat, Faizal Bux

**Affiliations:** 1 Institute for Water and Wastewater Technology, Durban University of Technology, Durban, 4000 South Africa; 2 School of Biological Sciences, University of East Anglia, Norwich Research Park, Norwich, NR4 7TJ UK; 3 Department of Analytical, Environmental and Forensic Sciences, Faculty of Life Sciences and Medicine, King’s College London, London, SE1 9NH UK

**Keywords:** Bioactive compounds, Algal biopesticides, Biological activities, Sustainability, Circular bioeconomy

## Abstract

The rapid growth in global population and the incessant demand for food warrant greater attention to sustainable agricultural practices. To ensure adequate food security, protection of crops from exposure to biotic agents (e.g., pathogens and insects) is imperative. Over the decades, synthetic pesticides have been used to control pests in agriculture. However, excessive and uncontrolled use of these chemical compounds causes serious environmental and health hazards. Therefore, algal biopesticides are used as biological agents to control soil-borne diseases and plant pests. The algae secrete a broad spectrum of metabolites, including phenolic compounds, terpenoids, alkaloids, peptides, and others. These bioactive compounds exhibit insecticidal, nematocidal, herbicidal, antibacterial, antifungal, and antiviral activities, thereby enhancing crop protection and productivity. Algal biopesticides are effective, eco-friendly, biocompatible, sustainable, and biodegradable, with low or no toxicity to the environment and beneficial off-target organisms. Thus, the present review focuses on the mechanisms of action and biological activities of various bioactive compounds produced from macroalgae and microalgae for exploitation as biopesticides. In addition, it presents the much-needed techno-economic analysis (TEA) and conceptual scenarios for cost-effective algal biopesticide production by integrating wastewater-based cultivation systems and designing multiple biorefinery approaches. The regulatory pathways for algal biopesticides, as well as some limitations hindering their commercial production, are also discussed.

## Introduction

The rise in global population growth and the incessant demand for food are worrisome, necessitating urgent attention to sustainable agricultural practices to ensure adequate food security (Poveda and Díez-Méndez 2023). Exposure of crops to biotic agents (e.g., pests and pathogens) results in significant agricultural losses. Consequently, plant diseases are recognized as a challenge to achieving global food security amid a growing human population (Velásquez et al. 2018; Mohammad-Razdari et al. 2022). The use of pesticides in agriculture is crucial for preventing, controlling, or destroying pathogens, insects, weeds, and other pests that affect plant growth (Costa et al. 2019; Ahmad et al. 2024). Pesticides are classified based on their sources, chemical structures, application methods, hazards, and mechanisms of action (Ahamad and Kumar 2023). They exist in both synthetic and biological forms. Synthetic pesticides are chemical substances used to control pests, with common classes including carbamates, organophosphates, synthetic pyrethroids, and organochlorines (Ahamad and Kumar 2023). The extensive and irrational use, overspray, and accidental spillage of these chemical substances have resulted in the widespread and persistent presence of pesticides in the environment, causing serious genotoxic, carcinogenic, and mutagenic effects on aquatic and terrestrial ecosystems and human health (Mohanty and Mohanty 2023a). In addition, chemical pesticides hurt soil biodiversity, reduce soil respiration, decrease macroorganism (e.g., earthworms) activity, and decrease plant photosynthetic activity and seed production (Farooq et al. 2019; Ayilara et al. 2023). Therefore, the development of sustainable, environmentally benign substitutes for synthetic pesticides is imperative.

Biopesticides are naturally derived substances from living organisms (e.g., plants, animals, and microbes) or their metabolites that suppress the growth and proliferation of plant pathogens and pests (Parmar et al. 2023). Compared to synthetic pesticides, these biocontrol agents are effective, target-specific, biocompatible, sustainable, and biodegradable, offering minimal environmental toxicity and lower pest resistance risks. They effectively function by inhibiting the growth, reproduction, or nutrition of phytopathogens or pests (Aioub et al. 2024). Additionally, biopesticides inhibit protein synthesis and disrupt plasma membrane permeability, ultimately resulting in cell death (Kumar et al. 2021). The worldwide market value of biopesticides was $5.75 billion (2022), and this is projected to reach $17.57 billion (2030) at a compound annual growth rate of 15% (Akmukhanova et al. 2023). However, biopesticides face high production costs, short shelf life, and limited field efficacy. Based on their sources, biopesticides are divided into microbial biopesticides, biochemical biopesticides, and botanical biopesticides. Microbial pesticides are produced from microbes, including bacteria, fungi, and algae. However, algae-based pesticides are prominent owing to the potential of the photosynthetic organisms to produce and accumulate a vast array of bioactive metabolites with potent and varying biological activities.

Algal biopesticides are biological agents used to control pathogens, pests, and soil-borne diseases in plants. Algae (including microalgae and macroalgae) secrete a plethora of metabolites, including polyphenols, tocopherols, oils, allelochemicals, sesquiterpenes, saponins, fatty acids, polysaccharides, and nitrogen-rich peptides, which are extracted and utilized to produce biopesticides (Sekar et al. 2025). These bioactive compounds exhibit insecticidal, nematocidal, herbicidal, and antimicrobial activities, thereby enhancing crop protection and productivity (Kumar et al. 2021; Mohanty and Mohanty 2023b). In addition, the metabolites inactivate enzymes, disrupt cytoplasmic membranes, and inhibit protein synthesis in targeted microbes or pests.

Biopesticide production from microalgae (e.g., *Chlorella vulgaris*, *Chlorella fusca*, *Chlorella sorokiniana*, *Tetradesmus obliquus*, *Scenedesmus* sp., and *Monoraphidium* sp.), cyanobacteria (including *Anabaena* sp., *Nostoc* sp., *Spirulina platensis*, and *Tolypothrix* sp.), and macroalgae (e.g., seaweeds) has been studied for sustainable pest management in agriculture (Costa et al. 2019; Ferreira et al. 2021; Hernández-Fernández et al. 2021; Jokel et al. 2023; Mohanty and Mohanty 2023a & b; Sekar et al. 2025). The broad biochemical characteristics of algae offer opportunities to discover new, highly bioactive compounds with a distinctive mode of action against pests and phytopathogens (Sekar et al. 2025). These organisms are a sustainable resource that can be harvested as a feedstock for producing biopesticides. However, the requirement for large amounts of nutrients and water for the mass cultivation of algae poses a significant challenge to the commercial production of algal biopesticides.

Algal bioactive compounds are a multi-functional and multi-target alternative to conventional pesticides, but their transition from promising laboratory agents to commercial biopesticides depends on cost optimization, regulatory clarity, and large-scale validation. Within the algal biorefinery field, numerous techno-economic analysis (TEA) studies have been reported for biofuel and bioplastic production, CO₂ sequestration, wastewater treatment, and pigment extraction (Bhatt et al. 2022; Figueroa-Torres and Theodoropoulos 2023). However, TEA studies focused specifically on biopesticide production from algal biomass remain scarce. Integrating biological properties and mechanisms with techno-economic and regulatory realities is imperative to ascertain the commercial viability of algal biopesticides. Thus, this review provides insights into the mode of action and biological activities of algal bioactive compounds for utilization as biopesticides. Additionally, it presents the TEA and conceptual scenarios for cost-effective algal biopesticide production by integrating wastewater-based cultivation systems and multiple biorefinery approaches. Furthermore, it discusses the regulatory frameworks for algal biopesticides and highlights the limitations that hinder their commercial production.

## Environmental and health effects of synthetic pesticides

Synthetic pesticides are chemical substances that are toxic to pests, phytopathogens, animals, humans, and the environment (Costa et al. 2019). These pesticides inhibit enzyme activity, modify the signaling system, and disturb electrolytic equilibrium. In addition, they degrade lipophilic membranes and generate free radicals, destroying organisms’ DNA, proteins, and tissue (Fang et al. 2020). Excessive and uncontrolled use of these compounds results in air, agricultural, and aquatic pollution, as well as food contamination (Cech et al. 2023).

Based on comparative toxicity studies, synthetic pesticides exhibit higher acute toxicity (lower lethal dose (LD)_50_). For instance, Vehedi et al. (2024) assessed the comparative toxicity of three insecticides, namely malathion, deltamethrin, and fenitrothion, on *Blattella germanica*, an urban pest. Using bioassay tests, after 1 h of exposure, malathion had the lowest LD_50_ of 4.29 ppm, making it more potent at lower concentrations. Acute toxicity of the biopesticide (neem gold) and two synthetic pesticides (permethrin and monocrotophos) was evaluated in juvenile *Cyprinus carpio* Linn at 96 h. The toxicity tests gave lethal concentration (LC)_50_ values of 75.49, 72.26, and 56.89 mg/L for neem gold, monocrotophos, and permethrin, respectively, suggesting the efficacy and toxicity of synthetic pesticides (permethrin followed by monocrotophos) (Davoodi and Abdi 2012).

According to a report by the United Nations Environment Programme (UNEP) and World Health Organization (WHO), 3 million people are exposed to pesticide poisoning, while 200,000 die globally, particularly in low- and middle-income countries (Ahmad et al. 2024; Desye et al. 2024). The increased exposure of humans to these substances has been linked to acute and chronic diseases, including endocrine disruption, neurotoxicity, type 2 diabetes, cancers, Parkinson’s Disease, Alzheimer’s disease, infertility, and leukemia (Foucault et al. 2021; Perrin et al. 2021; Rani et al. 2021; Frisoni et al. 2022; Hernández-Mariano et al. 2022; Rafeeinia et al. 2022; Wang et al. 2024). Lower amounts of synthetic pesticides that are applied to crops get to the target organisms, thereby causing greater threats to the sustainability of the ecosystems. The pesticide residues destroy natural vegetation, deteriorate surface and groundwater quality, and kill non-target organisms (e.g., livestock, bees, and wildlife) (Ahmad et al. 2024). In addition, these chemical agents leach into the subsoil and pollute groundwater. The incessant use of chemical pesticides permits greater resistance to pests, warranting the use of higher doses of the substances, which also pose greater toxic effects, revival, and epidemics of new pests. The unceasing and unrestricted use of synthetic pesticides leads to the accumulation of toxic chemicals in the soil, which harm the beneficial organisms (e.g., earthworms) and disrupt soil fertility. The contamination of water bodies by chemical pesticides poses significant hazards by reducing the dissolved oxygen levels required for aquatic organisms (e.g., fish) to survive. The quantitative data on the impacts of pesticide toxicity are shown in Table 1.

**Table 1:** Quantitative data on the effects of pesticide toxicity

Impact	Quantitative data	Reference
Air pollution	Pesticide was found about 100 km from the application site	Lavin and Hageman (2013)
Biodiversity	A decrease (~ 30%) in bee population	Goulson et al. (2015)
Soil	Decline (~ 30%) in soil microbial diversity	Zhang et al. (2017)
Water contamination	Pesticide detected in 50% of global water samples	Syafrudin et al. (2021)

## Algal metabolites with pesticidal properties

Algae are a prolific source of a broad spectrum of secondary metabolites, which are secreted in response to abiotic or biotic stresses within the ecosystems. These metabolites exhibit diverse biological activities, making algae an excellent biofactory in agrobiotechnology (Akmukhanova et al. 2023). The major classes of algae-derived bioactive compounds are elucidated in Table 2 and discussed in detail below.

**Table 2:** Major classes of algae-derived bioactive compounds linked to biopesticide function

Compound class	Representative example	Mode of action	Common source (algae type)	Reference
Fatty acids, free lipids	Linoleic, eicosapentaenoic acids	Membrane disruption, enzyme inhibition	Diatoms, microalgae	Costa et al. (2019); Casanova et al. (2023)
Phenolics/polyphenols	Flavonoids, phenolic acids	ROS induction, enzyme inhibition, and allelopathy	Macroalgae, green algae	Esserti et al. (2017); Jönsson et al. (2020); Asimakis et al. (2022)
Alkaloids/isoquinolines	Hapalindole	Neurotoxicity, ion channel modulation	Cyanobacteria, blue-green algae	Ansari et al. (2012); Poveda and Díez-Méndez (2023); Hamrouni et al. (2025)
Terpenoids/steroids	Squalene derivatives, diterpenes	Hormonal interference, membrane disruption	Macroalgae, brown algae	Radwan et al. (2011); Costa et al. (2019); Casanova et al. (2023)
Polysaccharides and sulfated sugars	Laminarin derivatives, carrageenans	Induction of host immunity	Macroalgae (brown, red)	O’Keeffe et al. (2019); Jönsson et al. (2020); Lu et al. (2021)
Small aldehydes/ketones	Volatile carbonyls, aldehydes	Oxidative stress, cell damage	Microalgae, macroalgae	Paulert et al. (2007); Ngala et al. (2016); Ramkissoon et al. (2017)

### Terpenoids

Terpenoids (also known as isoprenoids) constitute the largest class of bioactive natural products, comprising more than 50,000 identified compounds (Breitmaier 2006; Akmukhanova et al. 2023). Based on the number of isoprene units, they are classified as hemiterpenoids (1 isoprene unit, C5), monoterpenoids (2 isoprene units, C10), sesquiterpenoids (3 isoprene units, C15), diterpenoids (4 isoprene units, C20), sesterterpenoids (5 isoprene units, C25), triterpenoids (6 isoprene units, C30), tetraterpenoids (8 isoprene units, C40), and polyterpenoids (> 8 isoprene units, > C40). (Abdallah and Quax 2017; Singh et al. 2017). Structurally, terpenoids may occur as monocyclic, bicyclic, or linear molecules, depending on the number and arrangement of their cyclic components. These compounds are produced as secondary metabolites by numerous algal species, with red algae representing particularly prolific terpenoid producers (Wei et al. 2019). In algae, terpenoid biosynthesis occurs via the methylerythritol phosphate (MEP) and mevalonate (MVA) pathways. The MEP pathway occurs in the algal chloroplast by utilizing glyceraldehyde-3-phosphate and pyruvate for the synthesis of isopentenyl diphosphate and dimethylallyl diphosphate, serving as precursors for carotenoids and phytol. Conversely, the MVA pathway takes place in the cytosol of microalgae. In the presence of acetyl-CoA, it generates isopentenyl diphosphate, which is then converted to dimethylallyl diphosphate. This pathway provides precursors for sesquiterpenes and sterols (Bergman et al. 2019; Huang et al. 2021). Tetraterpenoids (carotenoids), including astaxanthin, zeaxanthin, and β-carotene, are especially abundant in phototrophic organisms, such as cyanobacteria, where they play essential roles in light harvesting and energy dissipation (Merhan 2017). Other terpenoids, such as comnostins and eremophilone, are produced by *Nostoc* sp. and *Calothrix* sp., respectively (Jaki et al. 2000; Höckelmann et al. 2009). Moreover, terpenoids exhibit a range of biological activities, including antioxidant and antimicrobial properties, making them valuable candidates for use as biopesticides in agriculture. Their application has demonstrated significant potential in protecting crops from phytopathogens and insects (Pavela and Benelli 2016). Sesquiterpenes and monoterpenes from extracts of *Cladophora* and *Chaetoceros* algae exhibited insect-repellent effects by acting on insect sensory organs (Thitame and Aher 2025).

### Alkaloids

Alkaloids are well-known allelochemicals with significant pesticidal properties. These bioactive compounds are nitrogen-rich heterocyclic molecules and are widely produced by dinoflagellates and cyanobacteria (Casanova et al. 2023). In algae, alkaloids occur predominantly as indole alkaloids, including velvitindolinones, ambigines, hapalindolinones, fishambigines, and hapalindoles (Walton and Berry 2016). However, the presence of hapalindole alkaloids and tjipanazoles contributes to the strong fungicidal activity observed in several cyanobacterial taxa (Vasas et al. 2010). Algae-derived alkaloids are promising natural biocides that induce severe oxidative stress in target pests, pathogens, and competing organisms. These compounds excessively produce reactive oxygen species (ROS), causing cell structural damage, DNA damage, and inducing programmed cell death in target pests (Rezayian et al. 2019; Karakoti et al. 2023). Additionally, these bioactive compounds exhibit potent herbicidal, insecticidal, and antibacterial activities against pests through various mechanisms, including cytotoxicity, induction of plant defense responses, and neurotoxicity (Asimikis et al. 2022; Refaay et al. 2025). However, alkaloids exhibit low toxicity and greater selectivity for beneficial organisms (Beev et al. 2025). Examples include 12-epi-hapalindole E isonitrile (*Fischerella* sp.), domoic acid (*Pseudo-nitzschia* sp.), calothrixin A (*Calothrix* sp.), and saxitoxin (diatoms) (Doan et al. 2000; Murray et al. 2011; Orr et al. 2013; Brunson et al. 2018).

### Phenolic compounds

Phenolic compounds are a broad category of compounds that contain at least one aromatic ring bonded to one or more hydroxyl (OH) groups. These bioactive compounds are grouped into polyphenols and phenols, with phenols subdivided into phenylpropanoids, flavonoids, and phenolic acids (Singh et al. 2017; Gonçalves 2021). The polyphenols are found mostly in seaweeds and exist as bromophenols, mycosporine-like amino acids, and phlorotannins. They offer several advantages over plant-derived phenolics, including higher antioxidant potency, a broad spectrum of biological activities, and high tolerance to environmental stress (Cichoński and Chrzanowski 2022). Algal phenolic compounds act as potent biopesticides by targeting structural and biochemical constituents in pests and pathogens, including plant defense pathways, metabolic enzymes, and cell membranes, as well as exhibiting high genotoxicity and cytotoxicity (Pereira et al. 2026). They exhibit strong antioxidant, antifungal, and antibacterial properties. Furthermore, polyphenols trigger plant defense mechanisms by increasing their resistance to microbial and rodent attacks (Sekar et al. 2025). Phenolic compounds play a crucial role in algal reproduction, growth, communication with interacting species, as well as protection from oxidative damage caused by ROS and OH radicals (Kumar and Goel 2019; Del Mondo et al. 2021). Additionally, phenolic compounds from *Gracilaria* and *Chlorella* extracts demonstrated repellent effects on mosquitoes and deterred egg-laying (Yuan et al. 2023).

The phenolic acids allow algae to adapt to environmental strains that can lead to DNA damage (Singh et al. 2014). They are synthesized via the phenylpropanoid pathway using phenylalanine or tyrosine as precursors. The enzymatic apparatus required for vital phenolic intermediates is found in all major divisions (Del Mondo et al. 2021). Algae, such as *Botryococcus braunii*, *Tetraselmis suecica*, *Isochrysis* sp., *Neochloris oleoabundans*, *Chlorella vulgaris*, *Chaetoceros calcitrans*, *Isochrysis galbana*, and *Phaeodactylum tricornutum*, have been investigated to produce phenolic compounds (Goiris et al. 2012; Cichoński and Chrzanowski 2022).

### Polyketides

Polyketides derived from algae are highly significant in the development of biopesticides due to their diverse chemical structures and potent bioactive properties. These compounds serve as a defensive mechanism against plant pathogens and pests by exhibiting antioxidant, herbicidal, insecticidal, larvicidal, and antimicrobial properties (Kizhakkekalam et al. 2020; Chakraborty et al. 2022; Beev et al. 2025). They are synthesized from carboxylic acid precursors via a series of condensation, reduction, and dehydration reactions, catalyzed by polyketide synthases (Casanova et al. 2023). They exist as type I, type II, and type III polyketides. They are a major class of secondary metabolites in algae, accounting for almost 25% of all well-known bioactive compounds from marine macroalgae (Sasso et al. 2012). Polyketides are characterized by high oxygen content and complex macrolide structure (Baerson and Rimando 2007). These bioactive compounds are usually found in cyanobacteria and toxic blooms (Sasso et al. 2012; Demay et al. 2019). A typical example is trichophycin A, a linear polyketide derived from *Trichodesmium* (Bertin et al. 2017; McManus et al. 2020). Other polyketides include amphidinins (*Amphidinium* sp.) and cryptophycins (*Nostoc* sp.) with potential antifungal and herbicidal activities (Casanova et al. 2023; Wang et al. 2023).

### Peptides

Peptides constitute a major class of algal allelochemicals and are produced through two distinct pathways, namely non-ribosomal peptide synthesis and ribosomal synthesis, followed by post-translational modification (Qoms et al. 2025). The non-ribosomal peptides are biochemically active compounds produced via an enzymatic complex referred to as non-ribosomal peptide synthetases, found in cyanobacteria (e.g., *Nostoc* sp.). Their biosynthesis involves sequential enzymatic reactions acting on a diverse set of precursor molecules (Nikolouli and Mossialos 2012; Akmukhanova et al. 2023). This group includes microcystins, which are produced by cyanobacterial genera, such as *Anabaena*, *Microcystis*, and *Oscillatoria*. In contrast, ribosomally synthesized peptides, including cyanobactins such as patellamide A and tenuecyclamide C, are found in *Lyngbya*, *Trichodesmium erythraeum*, *Prochloron*, and *Nostoc*, and exhibit notable antimicrobial activity (Sivonen et al. 2010; Micallef et al. 2014; Rojas et al. 2020). Additionally, algae-derived peptides possess insecticidal, nematocidal, and repellent properties (Park et al. 2011; Chanthini et al. 2024). Algal peptides exert antioxidant activity through ROS scavenging, inhibition of lipid peroxidation cascades, and direct metal chelation (Nimalaratne et al. 2015). A peptide from *Chlorella vulgaris* showed antioxidant capacity, found to be 26-fold higher (197 ng/mL Trolox equivalent) than Trolox after testing using the oxygen radical absorbance capacity (ORAC) test (Sheih et al. 2009). However, the stability of these compounds is a crucial challenge since they are sensitive to proteolytic degradation and environmental factors. This can be improved through encapsulation and chemical modifications (Pereira et al. 2019).

## Extraction of bioactive compounds from algae

The extraction of bioactive compounds from algae is imperative for the proper utilization of the secondary metabolites for a broad spectrum of applications, including the synthesis of biopesticides. The selection of suitable and optimal techniques for extracting bioactive compounds from algae is crucial to attain higher concentrations of desired bioactive compounds and obtain extracts devoid of toxic substances (Pereira et al. 2026). This is achieved using an array of techniques, including aqueous, microwave-assisted, ultrasound-assisted, enzyme-assisted, and supercritical extraction (Sekar et al. 2025). Some of the extraction methods are discussed below.

### Supercritical fluid extraction

The supercritical fluid extraction method is a modern and effective technique for extracting lipophilic compounds from algae. This technique uses CO₂ at a critical point above 30 °C and 7380 kPa as a supercritical fluid owing to its low toxicity, chemical stability, cost-effectiveness, and nonflammability (Saeed et al. 2024). It is suitable for recovering fatty acids, terpenoids, and other nonpolar compounds. Remarkably, Mehariya et al. (2019) used supercritical fluid extraction to isolate lutein from *Scenedesmus almeriensis*. In addition, this technique is environmentally benign, highly selective, requires low temperatures, generates no solvent residues, and maintains the integrity of thermostable molecules (Uwineza and Waśkiewicz 2020). In comparison to conventional extraction methods, supercritical fluid extraction requires less organic solvent, short extraction times, and offers high extract selectivity and compatibility with automation. However, the high cost of the equipment, coupled with significant expenses in its industrial application, surpasses its technical advantages (Putra et al. 2024). In addition, the efficiency of this technique is dependent on extraction duration, temperature, pressure, and target compound solubility (Hu et al. 2025).

### Enzyme-assisted extraction

Enzyme-assisted extraction involves the application of specific enzymes for the degradation of the algal cell wall, thereby enhancing the release of intracellular bioactive compounds in the cell. This approach is operated under mild conditions, thus preserving the structural integrity of the sensitive target compounds. However, further purification of the extracts is needed for the removal of residual enzymes and impurities (Lubek-Nguyen et al. 2022; Pereira et al. 2026). During the extraction process, little energy and low temperatures are used, thereby preventing degradation of sensitive bioactive compounds. This technique has been used for extracting many bioactive compounds, including allophycocyanin (*Arthrospira platensis*), polyphenols (*Ascophyllum nodosum*), carotenoids (*Ulva latuca*), and phlorotannins (*Sargassum dupplicatum*) from their respective algae (Tavanadi et al. 2019; Bio et al. 2020; Costa et al. 2022; Periaswamy Sivagnanam et al. 2024). In general, enzyme-assisted extraction is a green and sustainable technology for extracting bioactive compounds for potential industrial applications.

### Microwave-assisted extraction

The microwave-assisted extraction process requires microwave heat to enhance the extraction of bioactive compounds from algal biomass in the presence of suitable solvents. It is operated under mild, non-photolytic conditions that help preserve the structural and functional integrity of bioactive compounds (Gil-Martin et al. 2022). This technique is useful for the recovery of polyphenols and sulfated polysaccharides from algae. For instance, bioactive compounds possessing insecticidal and larvicidal properties were extracted from *Gracilaria* sp. using a microwave-assisted extraction method (Kumar et al. 2019). When compared to traditional extraction methods, this method is reproducible, high-throughput, rapid, energy-efficient, requires less solvent, and has a favorable temperature gradient to recover diverse bioactive compounds from algae. In addition, it offers uniform heating, efficient cell disruption, high yields of bioactive compounds, and shorter processing times (Maskur et al. 2023; Mungwari et al. 2025; Pereira et al. 2026). These characteristics qualify microwave-assisted extraction as a scalable and eco-friendly extraction strategy for the sustainable recovery of high-value algal metabolites.

## Mechanisms of action of algal bioactive compounds

**Fig. 1:**
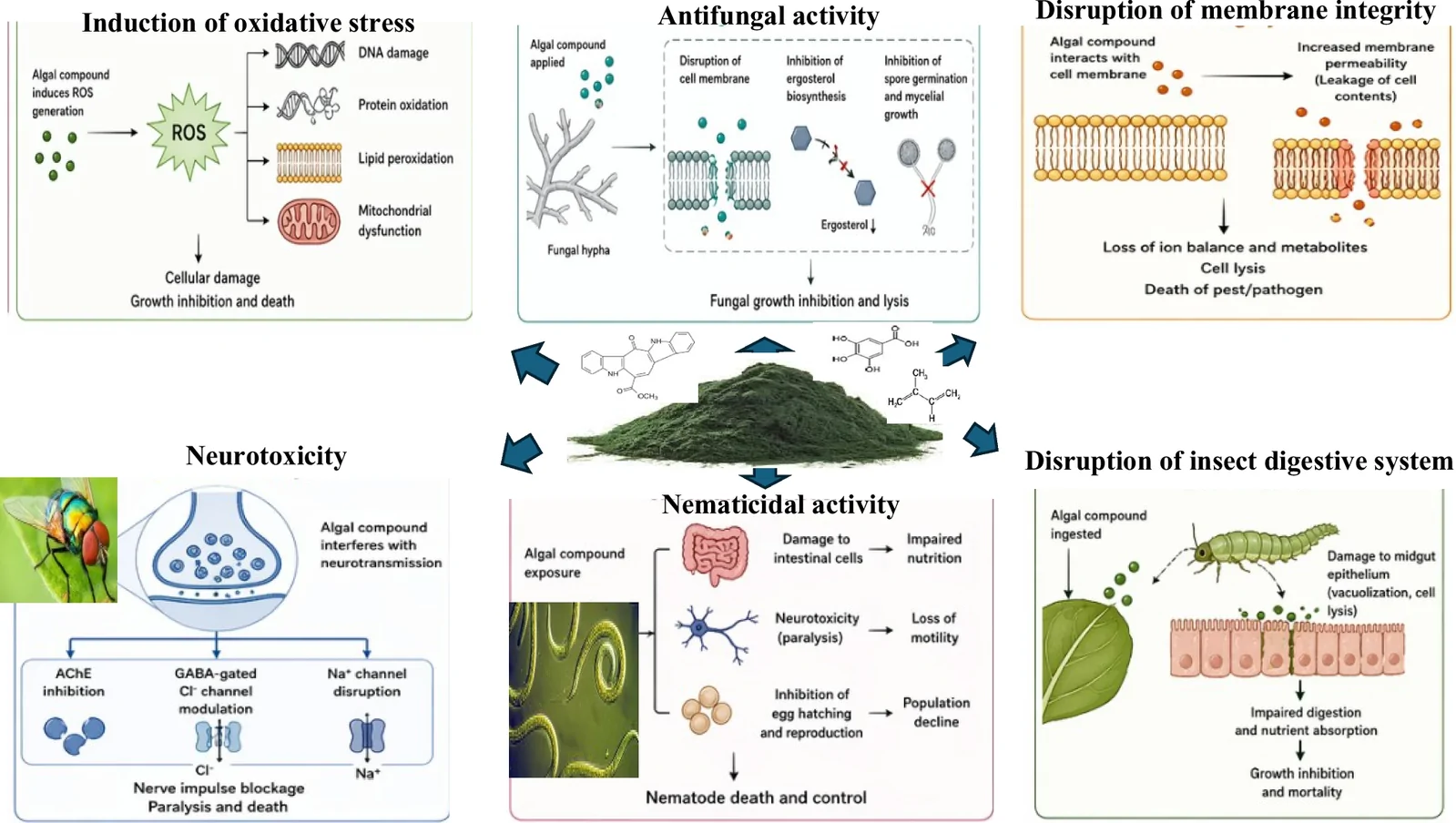
Schematic diagrams illustrating the mechanisms of action of algal bioactive compounds as biopesticides

Phototrophic organisms, including microalgae and macroalgae, have emerged as promising and environmentally sustainable biocontrol agents in agriculture due to their diverse biological activities. Their protective effects are mediated through multiple mechanisms, including the direct suppression of plant pathogen growth, the indirect activation of systemic plant resistance, and the modulation of soil microbial communities (Sadvakasova et al. 2025) (Table 3) (Fig. 1). In direct mechanisms of action, algal bioactive compounds function as biopesticides through multiple pathways, including the disruption of pest physiological processes (neurotoxicity, metabolic inhibition), antimicrobial action via cell membrane destruction, herbicidal activity through photosynthesis inhibition, and the stimulation of systemic plant immunity against pathogens (Shah et al. 2021; Akmukhanova et al. 2023; López-Arellanes et al. 2025).

**Table 3:** Mechanisms of action of bioactive compounds derived from algae

Algae	Bioactive compound	Mode of action	Pathogen/pest	Reference
*Ulva* sp.	Ulvan	Improved expression of salicylic acid-related genes	*Phytophthora parasitica* var. *nicotianae*	Cluzet et al. (2004)
*Pelvetia canaliculata*	Oligofucans	High activity of lipoxygenase and phenylalanine ammonia lyase	Tobacco mosaic virus	Klarzynski et al. (2000)
*Ulva fasciata*	Ulvan	Enhanced activity of glutathione, ascorbate peroxidase, NADPH oxidase, superoxide dismutase, and phenylalanine ammonia lyase	*Alternaria brassicicola*	de Freitas and Stadnik (2012)
*Sargassum* sp.	Unknown	Enhanced peroxidase, phenylalanine ammonia lyase, and glucanase activity	*Fusarium oxysporum* f. sp. *lycopersici*	Melo et al. (2020)
*Laminaria digitata*	Laminarin	Overexpression of salicylic acid-associated genes	*Erwinia carotovora*	Klarzynski et al. (2000)
*Bifurcaria bifurcata*	Alginate oligoalginates	Higher phenylalanine ammonia lyase activity	Biotic stress undetected	Aitouguinane et al. (2020)
*Scenedesmus obliquus*	Glucosamine	High secretion of diacetylene compounds	Biotic stress not reported	Stojakowska et al. (2008)
*Porphyridium sordidum*	Exopolysaccharides	Overexpression of salicylic acid-linked genes	*Fusarium oxysporum*	Drira et al. (2021)
*Dunaliella salina*	Exopolysaccharides	Enhanced phenolic profile	Biotic stress not detected	Arroussi et al. (2018)
*Chlorella fusca*	D-lactic acid	Overexpression of salicylic acid and jasmonic acid-associated genes	*Pseudomonas syringae*	Lee et al. (2020)
*Scenedesmus subspicatus*	Unknown	Higher antioxidant activity	Biotic stress not observed	Gemin et al. (2021)
*Porphorydium* sp.	Unknown	Enhanced steroidal glycoalkaloid content	Biotic stress not detected	Rachidi et al. (2020)
*Botryococcus braunii*	Unknown	Improved capsaicin and vanillylamine content	Biotic stress not reported	Sharma et al. (2010)
*Haematococcus pluvialis*	Unknown	Hyper-production of thiophene and betalaine	Biotic stress not observed	Rao et al. (2001)

In the indirect mode of action, algal bioactive compounds elicit systemic resistance in plants, activate defense enzymes, and enhance tolerance to biotic stress through pathways, including the phenylpropanoid pathway for strengthening cell walls, jasmonic acid and salicylic acid signaling, and the activation of antioxidant defenses to combat pathogen and pest attack (Chanthini et al. 2024). Algal metabolites suppress plant pathogens by stabilizing the structure and diversity of the rhizosphere microbiome and by enhancing nutrient cycling processes (Berthon et al. 2021; Shah et al. 2021). In some instances, algae form symbiotic associations with plants, leading to the release of bioactive compounds that activate plant defense pathways and promote systemic resistance to phytopathogens (Rachidi et al. 2021; Ouaddi et al. 2025).

Additionally, allelochemicals produced by microalgae, including flavonoids, antioxidants, alkaloids, phenols, peptides, and other metabolites, contribute to indirect protection by inhibiting the growth of a wide range of phytopathogens and functioning as natural herbicides and insecticides (Casanova et al. 2023; Righini et al. 2025). Algae also act as biopesticides through the secretion of secondary metabolites that inhibit photosynthetic processes in higher plants (Makepa et al. 2025). For example, cyanobacterin, produced by certain cyanobacteria, exhibits toxicity toward terrestrial angiosperms and aquatic *Lemna* species and disrupts photosynthetic electron transport in pea chloroplasts (Gleason 1986; Asimakis et al. 2022). Similarly, Fischerellin A, derived from *Fischerella muscicola*, inhibits photosynthetic electron transport in the aquatic macrophyte *Lemna minor* and suppresses the growth of several fungal pathogens, including *Pyricularia oryzae*, *Uromyces appendiculatus*, and *Phytophthora infestans* (Hagmann and Jüttner 1996). Therefore, algal biopesticides operate through an integrated molecular, microbial, and plant-mediated process, which collectively suppresses phytopathogens and enhances plant resilience.

Furthermore, some algal metabolites exhibit pesticidal effects by acting as potent enzyme inhibitors in plants. This is typical of microcystin-LR, nodularin, and oscillamide B and C, which are secondary metabolites secreted by cyanobacteria and inhibit protein phosphatase 1 (PP1) and protein phosphatase 2 A (PP2A). Both PP1 and PP2A are major regulatory enzymes that govern cellular processes, including gene expression, ion channel activity, and nitrogen and carbon metabolism (Toroser and Huber 2000). Anabenopeptins from algae inhibit the activity of zinc-containing carboxypeptidases A and B, thereby disrupting cell function and reproduction (Harada et al. 1995).

Bioactive compounds from algae can hamper the quorum-sensing (QS) system in plant pathogens. For instance, lignic acid (*Lyngbya mujuscula*), doscadenamide A, pitinoic acid A (marine cyanobacteria), and honaucins A, B, and C (*Leptolyngbya crossbyana*) have been reported to hinder the QS systems of pathogens, such as *Vibrio harveyi* and *Escherichia coli* (Montaser et al. 2011; Choi et al. 2012). QS inhibitors have also been discovered in *Chlorella saccharophila* and *Chlamydomonas reinhardtii* (Teplitski et al. 2004; Natrah et al. 2011). The insecticidal properties of halogenated monoterpenes from *Plocamium telfairiae* (red alga), telfairine and aplysiaterpenoid A resulted in 80% and 60% deaths in German cockroaches, respectively (Watanabe et al. 1990). Microalgal (*Prasiola crispa*) extracts have been reported to exhibit insecticidal activities in *Nauphoeta cinerea* (cockroach) and fruit fly (*Drosophila melanogaster*) (Zemolin et al. 2014).

## Biological properties of algal bioactive compounds

Macroalgae and microalgae represent an expanding frontier in biopesticide development due to their capacity to synthesize diverse secondary metabolites with potent bioactivities (Poveda and Díez-Méndez 2023). Beyond their well-established functions as biofertilizers and biostimulants, many algal taxa, including cyanobacteria, green microalgae, red and brown seaweeds, produce compounds with antifungal, antibacterial, antiviral, nematocidal, insecticidal, and herbicidal properties (Sithole et al. 2023). These bioactivities underpin their emerging role as environmentally benign alternatives to synthetic agrochemicals (Gonçalves 2021; Bhattacharjee 2022). Many species produce a wide range of secondary metabolites with antimicrobial, antifungal, and antiviral properties. These compounds can inhibit the germination of fungal spores, suppress the growth of phytopathogenic bacteria, and reduce the spread of viral diseases in crops (Asimakis et al. 2022).

Certain cyanobacteria also produce allelopathic compounds that disrupt pest development and reproduction, thereby contributing to natural crop protection (Chaïb et al. 2021). Overall, the integration of microalgae- and cyanobacteria-based biopesticides into pest management programs offers a sustainable approach to reducing reliance on chemical pesticides while enhancing agroecosystem resilience. Incorporating these algae-derived products into crop protection frameworks not only minimizes ecotoxicity but also supports long-term agricultural sustainability (Verma et al. 2024). Recent literature underscores the significant promise of algae-based biopesticides as eco-friendly alternatives to synthetic agrochemicals (Gonçalves 2021; Casanova et al. 2023; Mohanty and Mohanty 2023a,b; Sharanappa et al. 2024; Verma et al. 2024). The biological activities of selected algal bioactive compounds are summarized in Table 4 and discussed in detail below.

**Table 4:** Biological activities of some bioactive compounds derived from algae

Algae	Bioactive compound/extract	Biological activity	Reference
*Chlorella vulgaris*	Methanol extract	Antifungal	Dinev et al. (2021)
*Anabaena laxa*	Laxaphycin B & C	Antimicrobial	Frankmölle et al. (1992)
*Sargassum* sp.	Alkaloids	Nematicides	Zhou et al. (2024)
*Anabaena cylindrica*	Balticidins	Antifungal	Bui et al. (2014)
* Dictyota bartayesiana*	Fucoidan	Antiviral	Sanniyasi et al. (2019)
*Nostoc* sp.	Cryptophycin	Herbicidal	Biondi et al. (2004)
*Gracilaria* sp.	Sulfated polysaccharides	Insecticides	Kumar et al. (2019)
*Ulva lactuca*	Lectins	Fungicides	Mishra et al. (2020)
*Fucus spiralis*	Essential oil	Insecticidal	Boutjagualt et al. (2022)
*Microcystis aeruginosa*	Microcystin	Herbicidal	Berry et al. (2008)
*Cylindrospermum stagnale*	Cylindrofridins	Antibacterial	Preisitsch et al. (2016)
*Microcystis aeruginosa*	Kawaguchipeptins A & B	Antibacterial	Parajuli et al. (2016)
*Synechocystis aquatilis*	Norharmane	Herbicidal	López-González et al. (2020)
*Schytonema hofmanni*	Cyanobacterin	Herbicidal	Gleason and Case (1986)
*Plocamium cartilagineum*	Mertensene	Insecticidal	Argandoña et al. (2000)
*Caulerpa racemosa*	Alkaloid caulerpin	Insecticidal	Alarif et al. (2010)
*Laurecia papillosa*	Acetogenin	Insecticidal	Abou-elnaga et al. (2011)
*Laurecia nidifica*	Laurinterol	Repellent	Ishii et al. (2017)
*Ulothrix* sp.	Methanol extract	Antifungal	Zhao et al. (2025)
*Chaetomorpha antennina*	Methanol extract	Insecticidal	Chanthini et al. (2021)
*Chlamydomonas reinhardtii* strains, *Chlorella* strains, and *Chloroidium saccharophilum*	Fatty acids, isoquinoline, alkaloids, aldehydes, and cinnamic acids	Herbicidal and nematocidal	Brito-Bello and Lopez-Arredondo (2023)
*Oscillatoria* sp. SS-8	Phenolic compounds, tannins, saponins, and alkaloids	Antifungal	Seiilbek et al. (2025)

### Antibacterial activity

Although the antibacterial potential of algal extracts has been widely reported (Garuba 2026; Mandal et al. 2026; Sendekie et al. 2026), it is important to distinguish between studies conducted on agriculturally relevant phytopathogens and those using model microorganisms such as *E. coli* and *Listeria monocytogenes*. While the latter are commonly used for preliminary bioactivity screening, their ecological and physiological differences from plant pathogens limit direct extrapolation to crop protection applications. Therefore, such findings should be interpreted as indicators of antimicrobial potential rather than definitive evidence of biopesticidal efficacy.

The antibacterial efficacy of algal extracts has been well documented, particularly against phytopathogenic bacteria such as *Agrobacterium tumefaciens*, *Xanthomonas campestris*, *Erwinia carotovora*, and *Pseudomonas syringae* (Vicente et al. 2022; Ajuna et al. 2024). Numerous algal species produce antimicrobial compounds, including phenolic acids, polyunsaturated fatty acids, halogenated hydrocarbons, and cyclic peptides that compromise bacterial cell integrity and metabolism (Costa et al. 2019; Casanova et al. 2023). These algal metabolites disrupt bacterial cell membranes, inhibit QS, and interfere with enzyme activity (Costa et al. 2019; Mol and Purushothaman 2020). Antibacterial potency varies widely depending on compound class, extract composition, and target organisms. For example, extracts from *Chlorella vulgaris* and *Anabaena laxa* demonstrated strong inhibition zones against Gram-negative pathogens, attributed to lipid peroxidation and cell wall lysis (Casanova et al. 2023). Alsenani et al. (2020) studied the antibacterial potential of 20 microalgal strains against Gram-positive and Gram-negative bacteria. Among them, *Isochrysis galbana*, *Chlorella* sp. FN1, and *Scenedesmus* sp. NT8c demonstrated antibacterial activity against *Listeria monocytogenes* at concentrations ranging from 100 to 1000 µg/mL. They further characterized the antibacterial compounds and found the presence of linolenic acid, oleic acid, docosahexaenoic acid, and eicosapentaenoic acid in the crude extract. While not a plant pathogen, such assays provide preliminary insights into the spectrum and potency of algae-derived compounds. *Listeria monocytogenes* is a human pathogen but an opportunistic pathogen that can survive in vegetation, water, and soil (Truong et al. 2021). A similar study was carried out by Marrez et al. (2021) using *Oscillatoria* sp. The authors performed different solvent-based extractions to extract antibacterial compounds, which were tested against *Pseudomonas aeruginosa* and *Aspergillus flavus*. The diethyl ether extract showed antimicrobial activity against the tested organisms, *Pseudomonas aeruginosa* and *Aspergillus flavus,* with minimum inhibitory concentration (MIC) values of 20.7 and 20.2 mm, respectively. Similarly, Bhuyar et al. (2020) assessed the antibacterial activity of *Oscillatoria* sp. The microalgal crude extract at a concentration of 75–100 mg/mL inhibited the growth of *Pseudomonas aeruginosa*. Ambrico et al. (2020) evaluated the antibacterial activity of *Dunaliella salina* extracts against plant pathogens. Their in vitro tests showed that the hexane extract exhibited strong antibacterial activity, and further characterization identified β-carotene as a major component. In vivo assays on young tomato plants infected with *Pseudomonas syringae* showed a 65.7% reduction in disease incidence and a 77% reduction in severity. Similarly, treatment of tomato and zucchini fruits infected with *Pectobacterium carotovorum* subsp. *carotovorum* reduced soft rot symptoms to 5.3% and 12.6%, compared to 90.6% and 100% in the positive controls.

**Fig. 2:**
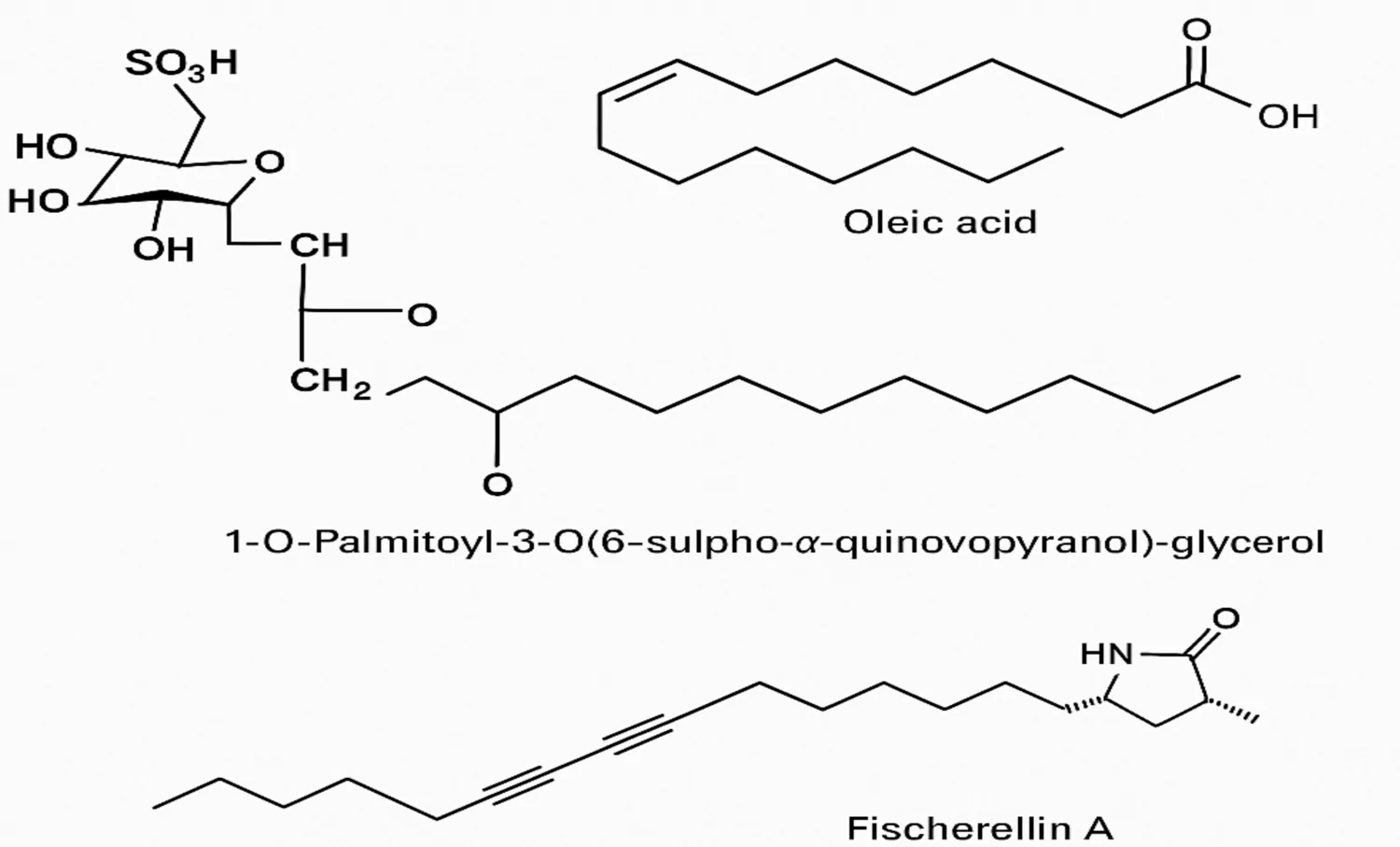
Chemical structures of selected algae-derived biopesticidal metabolites. Oleic acid represents unsaturated fatty acids that can disrupt microbial membranes; 1-O-palmitoyl-3-O(6′-sulpho-α-quinovopyranosyl)-glycerol illustrates sulfated glycolipids associated with antibacterial activity and plant defence elicitation; and Fischerellin A represents cyanobacterial secondary metabolites with inhibitory activity against photosynthetic and microbial targets

Moreover, polysaccharides from brown marine algae such as *Sargassum* sp. induce systemic resistance in plants, enhancing defense enzyme activity and phenolic content (Gonçalves 2021). The compound responsible for this action in *Sargassum wightii* is sulphoglycerolipid 1-O-palmitoyl-3-O(6’-sulpho-α-quinovopyranosyl)-glycerol (Fig. 2). Examples of algal extracts with antibacterial action are shown in Table 5. Brown algal polysaccharides, such as laminarin and fucoidan, also function as plant immunity elicitors, enhancing host defense enzyme activities, including peroxidase and phenylalanine ammonia-lyase (Gonçalves 2021). These systemic effects provide a dual antibacterial mechanism: direct pathogen inhibition and indirect plant immune priming.

**Table 5:** Antibacterial activities of some algal bioactive compounds

Algae	Extract or compound	Target organism	Disease/pathogen	Protected plant	Mode of action	Reference
*Cystoseira myriophylloides, Fucus spiralis*	Aqueous extracts	*Agrobacterium tumefaciens*	Crown gall disease	*Solanum lycopersicum*	Unknown	Esserti et al. (2017); O’Keeffe et al. (2019)
*Gracilaria edulis, Sargassum wightii,* and *Enteromorpha flexuosa*	Petroleum ether extracts, methanolic extracts, unsaponified and lipophilic fractions	*Xanthomonas oryzae*	Bacterial blight	Rice plants	Unknown	Umar and Rengasamy (2000)
*Sargassum wightii*	Sulphoglycerolipid (Methanol extract)	*Xanthomonas oryzae*	Bacterial blight	Rice plants	Unknown	Arunkumar et al. (2005)
*Sargassum wightii*	Methanolic extracts	*Pseudomonas syringae*	Leaf spot disease	*Gymnema sylvestre*	Unknown	Kumar et al. (2008)
*Ulva fasciata*	Methanolic extracts	*Xanthomonas campestris*, *Erwinia carotovora*	Plant pathogens	Various plants species	Unknown	Paulert et al. (2007)
*Sargassum latifolium, Hydroclathrus clathratus, Padina gymnospora*	Methanolic extracts	*Ralstonia solanacearum, Pectobacterium carotovorum*	Brown rot disease	Potato plants	Induction of plant defenses, formation of bioactive secondary metabolites	Ibraheem et al. (2017)
*Ulva lactuca, Gelidium serrulatum*	Alkaline extracts	*Xanthomonas vesicatoria*	Plant pathogen	Tomato plants (in vitro)	Induction of plant defenses	Ramkissoon et al. (2017)

Although algal extracts are often described as broad-spectrum antibacterial agents, their activity spectrum remains incompletely resolved because many studies test only a limited number of organisms under varying experimental conditions. Importantly, resistance development has rarely been systematically investigated. The presence of multiple bioactive compounds within algal extracts, targeting membranes, QS pathways, oxidative stress responses, and enzymatic systems, may reduce the likelihood of rapid resistance emergence compared to single-target antibiotics. However, this hypothesis remains largely untested, as few studies have conducted serial-exposure experiments or evaluated adaptive responses in target pathogens. Consequently, the long-term sustainability of algae-derived antibacterial agents requires further investigation. Additionally, in the above discussion, in vitro MIC values provide a baseline for antimicrobial potential; the transition to field-scale biopesticide application faces challenges such as photodegradation by ultraviolet (UV) radiation, microbial degradation in the soil, and poor rainfastness on the plant surface. However, the success of in vivo trials on tomato and zucchini suggests that these metabolites can maintain efficacy under simulated agricultural conditions (Ambrico et al. 2020). However, more large-scale field trials need to validate these laboratory outcomes.

### Antiviral activity

**Fig. 3:**
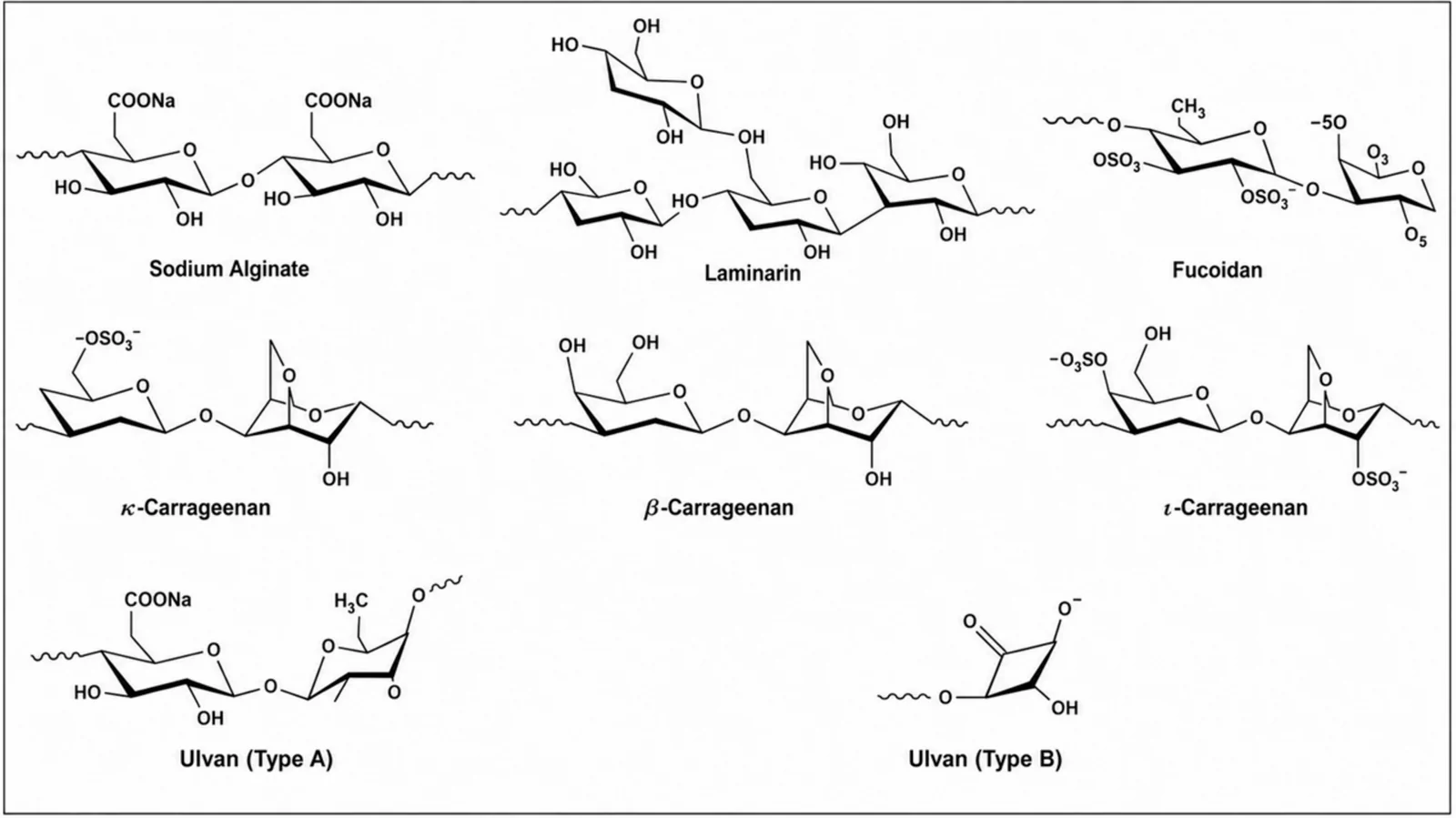
Structural representations of key antiviral polysaccharides derived from marine algae. Shown are the chemical structures of sodium alginate, laminarin, fucoidan, κ-carrageenan, β-carrageenan, ι-carrageenan, and ulvan, the major sulfated and non-sulfated polysaccharides implicated in the antiviral activity of algal biopesticides. These polymers exhibit diverse glycosidic linkages and degrees of sulfation that contribute to their broad-spectrum antiviral properties, including inhibition of viral adsorption, entry, and replication

Marine algae are a major source of structurally diverse sulfated polysaccharides, such as carrageenans, alginates, fucoidans, galactans, laminarins (Fig. 3), and various microalgal exopolymers, which exhibit potent broad-spectrum antiviral activity (Jönsson et al. 2020; Tiwari et al. 2022). These compounds act as natural biopesticides by inhibiting plant and animal viruses, making them promising agents for sustainable disease management (Ansari et al. 2012; Sarwar 2015). Several studies have shown that these compounds inhibit viral adsorption and replication by interfering with viral envelope glycoproteins (Costa et al. 2019; Casanova et al. 2023). The antiviral activity of algal polysaccharides is primarily attributed to their anionic sulfate groups, high molecular weight, and complex carbohydrate architectures (Lu et al. 2021). According to studies, these molecules consistently block the initial phases of viral infection, including viral adsorption to host cells (the dominant mechanism), inhibition of viral internalization, suppression of viral replication, DNA polymerase, or reverse transcriptase activity, and interference with viral envelope glycoproteins (Ahmadi et al. 2015). In plant systems, such molecules have been shown to protect against cucumber mosaic virus and tobacco mosaic virus through the elicitation of plant immunity pathways. The antiviral efficacy of *Spirulina platensis* and *Ulva lactuca* extracts has also been demonstrated in enhancing resistance and reducing viral symptom severity (Verma et al. 2024).

Overall, marine algal polysaccharides represent promising antiviral candidates, particularly because their sulfated groups and high molecular-weight structures can interfere with viral adsorption, entry, and replication. However, their agricultural relevance remains less established than their in vitro antiviral activity. Their performance in plant systems will depend on formulation stability, persistence on leaf surfaces, resistance to UV degradation and rainfall, and compatibility with existing virus-management strategies, such as resistant cultivars, vector control, and sanitation. Therefore, future studies should compare algal polysaccharides with existing antiviral or virus-management products under greenhouse and field conditions.

### Antifungal activity

Antifungal properties of algal metabolites have been widely recognized against major phytopathogens, including *Botrytis cinerea*, *Fusarium oxysporum*, and *Phytophthora infestans* (Shah et al. 2021; Bhardwaj et al. 2025). Active compounds include phlorotannins and phenolic acids, which disrupt fungal membranes; fatty acids and aldehydes, which impair mitochondrial function and ergosterol biosynthesis; and cyclic peptides from cyanobacteria that inhibit protein synthesis. Algal bioactive compounds exhibit promising antifungal activity in vitro and in controlled environments and are increasingly being explored as sustainable alternatives to synthetic fungicides (Lu et al. 2021; Shah et al. 2021). Phenolics, phlorotannins, and fatty acids from brown and red algae also exert fungicidal effects by damaging cell membranes, inhibiting spore germination, and interfering with ergosterol biosynthesis (Mol and Purushothaman 2020; Casanova et al. 2023).

The transition from laboratory potential to agricultural utility is supported by consistent performance across various application methods. For instance, extracts from marine macroalgae and microalgae prepared with solvents such as water, methanol, acetone, ethanol, chloroform, ethyl acetate, and benzene have demonstrated strong inhibitory effects against a wide range of phytopathogenic fungi (O’Keeffe et al. 2019; Jönsson et al. 2020). Cyanobacteria, such as *Nostoc* sp., produce metabolites capable of suppressing multiple fungal pathogens, with ethanolic extracts completely inhibiting *Sclerotinia sclerotiorum* in tomato plants (Esserti et al. 2017). The efficacy of these extracts demonstrates that bioactive metabolites can maintain functionality despite the complex chemical environment of the plant phyllosphere. Furthermore, aqueous extracts of brown algae, including *Cystoseira myriophylloides*, *Fucus spiralis*, and *Laminaria digitata*, demonstrate significant protection of tomato seedlings against *Verticillium dahliae* infection when applied as foliar sprays or drenches (Ibraheem et al. 2017). These diverse application methods (sprays vs. drenches) highlight the ability of algal compounds to operate across different agricultural matrices, including soil and leaf surface.

Organic extracts from *Lessonia innamomic* reduced necrotic lesion formation caused by *Botrytis cinerea.* In contrast, red algal extracts (e.g., *Gracilaria chilensis*) suppressed *Phytophthora cinnamomi* in a dose-dependent manner (Kumar et al. 2008). Brown seaweeds, such as *Sargassum latifolium* and *Padina gymnospora*, also exhibit significant antifungal activity against *Fusarium solani* and *Rhizoctonia solani* (Ansari et al. 2012).

The functional success of these extracts is deeply linked to their biochemical composition and dual-action mechanisms. Fatty acids, including palmitic acid, linoleic acid, and arachidonic acid, often present in high abundance, exert direct fungitoxic effects and can inhibit spore germination in pathogens such as *Phoma tracheiphila* (Paulert et al. 2007; Sarwar 2015). Beyond direct toxicity, polysaccharides, including laminarin, fucoidan, and alginate, also play key roles as immunity elicitors. Laminarin from *Laminaria digitata* stimulates host defense responses and protects grapevine and tomato plants against *Botrytis cinerea* and *Plasmopara viticola* (Paulert et al. 2007; O’Keeffe et al. 2019). Similarly, polysaccharides from *Anabaena*, *Ecklonia*, and *Jania* species inhibit *Botrytis cinerea* in strawberries (Jönsson et al. 2020), while ulvan, a sulfated heteropolysaccharide from *Ulva* sp., reduces the severity of *Colletotrichum gloeosporioides* infection in apple seedlings by enhancing peroxidase activity and activating defense pathways (Shah et al. 2021). Nevertheless, direct comparison with commercial fungicides remains limited, and standardized greenhouse and field trials are needed before practical replacement claims can be made.

Recently, microalgae have gained increasing attention due to their rich diversity of bioactive secondary metabolites. Various microalgal compounds, such as carotenoids, phenolic compounds, and fatty acids, demonstrated significant antifungal activity (Eckstein et al. 2024; Lage et al. 2024). Research by Senousy et al. (2022) identified two microalgal strains from soil and brackish water habitats in Egypt and studied their antifungal potential against phytopathogenic fungi. Their results showed that among the isolated microalgae, *Anabaena* sp. HSSASE11 (83.94%) showed the highest inhibition percentage against three fungal pathogens, *B. theobromae*, *F. solani*, and *P. ultimum*, respectively, followed by *Dunaliella* sp. HSSASE13 (81.94%) and *Oscillatoria nigroviridis* HSSASE 15 (63.42%). The authors suggested that flavonoids and phenolic compounds in microalgae are the major contributors to antifungal activity. A recent report by Eckstein et al. (2024) shows that different species of the microalga *Desmodesmus* demonstrate good antifungal activity, with inhibition effectiveness values ranging between 1.27 and 2.24. Given the growing trend of using microalgae as antifungal agents, Horvath et al. (2023) also isolated 280 microalgal strains (including Cyanophyceae, Chlorophyceae, Trebouxiophyceae, Klebsormidiophyceae, and Zygnematophyceae), and prepared water extracts, which were tested against six phytopathogenic fungi and three oomycetes. Overall, 45% of the strains inhibited at least one pathogen. Cyanobacteria showed the highest activity (64%), followed by Chlorophyceae (49%) and Trebouxiophyceae (30%). Nineteen strains exhibited fungicidal or oomyceticidal activity, mainly Cyanobacteria, which also had a broader spectrum of inhibition. Notably, *Trichormis variabilis* MACC-304 and *Tolypothrix tenuis* MACC-205 inhibited six pathogens, while *Nostoc linckia* MACC-612 inhibited four. Chlorophyta strains were generally active against only 1 to 2 pathogens. In general, these findings highlight algae as a rich source of antifungal agents that act directly on pathogens or indirectly through the induction of plant immunity. Their biochemical diversity, biodegradability, and agricultural efficacy position algal extracts as strong candidates for next-generation biopesticides. Despite these promising findings, many studies remain limited to controlled laboratory or greenhouse conditions. Environmental factors such as UV exposure, rainfall, microbial degradation, and variability in plant surfaces can significantly influence the stability and efficacy of algae-derived compounds in field settings. Therefore, further research is required to assess formulation stability, delivery mechanisms, and large-scale performance under agricultural conditions.

In conclusion, while the majority of existing research has been conducted under controlled laboratory conditions, these laboratory-derived MIC values provide an essential baseline for assessing antimicrobial potential. However, the true agricultural efficacy of algal extracts is driven by a dual mechanism: direct pathogen toxicity and indirect plant immune priming. Transitioning from these laboratory findings to large-scale greenhouse and field studies is critical for validating real-world performance.

Although large-scale reports remain limited, successful field and greenhouse trials have already demonstrated significant disease reduction in crops such as strawberries and tomatoes (Gonçalves 2021). These results indicate a strong potential for incorporating algal extracts into integrated pest management (IPM) systems. Furthermore, the systemic induction of resistance offered by these extracts helps overcome common environmental challenges, such as UV-induced photodegradation and soil adsorption, that typically limit the field performance of traditional, direct-contact biopesticides. By bridging the gap between in vitro data and in vivo application, algal metabolites emerge as highly promising candidates for sustainable, real-world agricultural solutions.

### Nematocidal activity

Marine and freshwater algae exhibit strong nematocidal potential. Phlorotannins, alkaloids, sulfated polysaccharides, and fatty acids from species such as *Sargassum*, *Gracilaria*, *Nostoc*, and *Anabaena variabilis* demonstrate nematocidal activity against root-knot nematodes (*Meloidogyne* sp.) and free-living nematodes (Hamrouni et al. 2025). These algae produce metabolites capable of immobilizing or killing plant-parasitic nematodes (Table 6). Additionally, microalgae, such as *Anabaena variabilis* and *Nostoc* sp., enhance soil microbial diversity, thereby indirectly suppressing nematode proliferation (Verma et al. 2024). However, the activity values summarized in these tables should be interpreted cautiously because testing conditions are not standardized across studies. Differences in algal species, extraction solvent, compound purity, exposure time, target-organism stage, and assay endpoint can strongly influence reported potency. Therefore, the tables are intended to identify promising compound classes and evidence gaps rather than provide a definitive ranking of efficacy.

**Table 6:** Nematocidal activities of some algal bioactive compounds, extracts, or products

Algae	Compound/type of extract/product	Test organism	Protected plant/organism	Mode of action	Reference
*Ascophyllum nodosum*	Commercial Formulation Algaefol® (Chema Industries, Egypt)	*Radopholus similis*, *Meloidogyne incognita*, *Belonolaimus longicaudatus*	Citrus, tomato, centipede grass	Cytotoxic effect	Morgan and Tarjan (1980); Radwan et al. (2011); Radwan et al. (2012)
*Ascophyllum nodosum, Ecklonia maxima*	Commercial Formulations Kelpak (Kelp Products Ltd., Simon’s Town, South Africa), OSMO® (OSMO® International NV, Diksmuide, Belgium)	*Meloidogyne chitwoodi*, *Meloidogyne hapla*	Tomato plants (*Lycopersicon esculentum*)	Interrupt enzymatic activities of hatching process, alter sensory perception of the roots by the nematodes	Ngala et al. (2016)
*Ecklonia maxima*	Commercial Formulation Kelpak 66 liquid concentrate (cancelled product)	*Meloidogyne incognita*	Tomato plants (*Lycopersicon esculentum*)	Unknown	Featonby-Smith and Van Staden (1983)
*Stoechospermum polypodioides*	Methanolic extracts	*Meloidogyne javanica*	In vitro	Unknown	Rizvi and Shameel (2006)
*Jania rubens*	Brominated diterpenes	*Allolobophora caliginosa*	In vitro	Unknown	Awad (2004)
*Spatoglossum variabile, Stokeyia indica, Melanothamnus afaqhusainii*	Dry powders	*Meloidogyne incognita*	Eggplant, watermelon	Direct cytotoxic effect on plant metabolism/resistance to stress	Baloch et al. (2013)
*Spatoglossum variabile, Melanothamnus afaqhusainii, Halimeda tuna*	Aqueous and ethanolic extracts	*Meloidogyne javanica*	Sunflower, tomato	Induction of plant defenses	Sultana et al. (2012)
*Nostoc* sp.	Methanolic extracts	*Caenorhabditis elegans*	In vitro	Induction of plant defenses	Biondi et al. (2004)

Early evidence demonstrated that brominated diterpenes isolated from the red alga *Jania rubens* exert potent nematocidal effects, effectively suppressing *Allolobophora caliginosa* (Jurado-Flores et al. 2025). Cyanobacteria also play a prominent role: methanolic extracts of *Nostoc* ATCC 53789 caused significant mortality and developmental delay in *Caenorhabditis elegans* (*C. elegans*), confirming the presence of metabolites capable of interfering with nematode growth and reproductive biology (Ansari et al. 2012). Such findings highlight the broad taxonomic range of algae capable of producing nematocidal compounds. Macroalgae have been particularly effective against root-knot nematodes (*Meloidogyne* sp.), which are among the most damaging agricultural pests worldwide. Dry powders from *Spatoglossum variabile*, *Stokeyia indica*, and *Melanothamnus afaqhusainii* reduced gall formation and inhibited penetration of *Meloidogyne incognita* into the roots of eggplant and watermelon, demonstrating both protective and curative effects (Poveda and Díez-Méndez 2023). Similar activity against *Meloidogyne javanica* has been reported in okra, sunflower, and tomato using aqueous and ethanolic extracts of *Sargassum tenerrimum*, *S. swartzii*, *S. wightii*, *Spatoglossum variabile*, *Melanothamnus afaqhusainii*, and *Halimeda tuna* (Aioub et al. 2024). In several cases, algal extracts achieved suppression levels comparable to the commercial nematicide carbofuran, and synergistic increases in efficacy were observed when *Spatoglossum variabile* extracts were applied in combination with the synthetic compound (Poveda and Díez-Méndez 2023).

Screening studies across multiple macroalgal species have further identified *Stoechospermum polypodioides* as one of the most potent nematocidal agents, causing almost 80% mortality in *Meloidogyne javanica*, the highest activity among twenty-one species evaluated (Sekar et al. 2025). Additional experiments have reported reduced root-knot nematode infestation in tomato following treatment with diverse macroalgal extracts, underscoring the consistency of these biopesticidal effects (Izydorczyk et al. 2025). Commercial macroalgal products also contribute to nematode management. Extracts from *Ascophyllum nodosum* and *Ecklonia maxima*, widely used as biostimulants, have demonstrated activity against *Meloidogyne chitwoodi* and *M. hapla*, primarily by disrupting egg hatching and sensory perception. Notably, alkaline extracts of *A. nodosum* consistently outperformed those from *E. maxima*, which in some cases inadvertently increased nematode infectivity (Sekar et al. 2025). This highlights the significance of the extraction method, chemical characterization, and product formulation in optimizing nematocidal efficacy. Taken together, these studies demonstrate that algae and cyanobacteria produce a chemically rich spectrum of nematocidal metabolites that target multiple developmental stages of plant-parasitic nematodes. Their ability to reduce galling, inhibit root invasion, impair egg hatching, and cause direct mortality supports the integration of algal nematocidal compounds into sustainable and environmentally compatible nematode management programs.

### Insecticidal activity

Algae are a rich source of insecticidal and acaricidal compounds, making them valuable candidates for environmentally sustainable pest management. Marine macroalgae and microalgae produce diverse bioactive metabolites (Cadar et al. 2025; Monroy-García et al. 2025). These include polysaccharides, phenolics, proteins, terpenoids, alkaloids, fatty acids, and halogenated compounds that act through multiple biochemical pathways to suppress arthropod pests (Sithole et al. 2023; Tran et al. 2024; Izydorczyk et al. 2025).

Unlike synthetic insecticides, which often rely on single-site toxicity, algae-derived metabolites exhibit multimodal mechanisms, thereby reducing the likelihood of resistance development (Poveda and Díez-Méndez 2023; Hamrouni et al. 2025). Algae-derived insecticides exhibit activity against a range of agricultural pests, including *Spodoptera litura*, *Aedes aegypti* larvae, aphids, and stored-grain beetles. Compounds responsible for insecticidal effects include terpenoids, sterols, peptides, polyphenols, and volatile aldehydes (Mol and Purushothaman 2020; Casanova et al. 2023). Mechanisms of insecticidal action include disruption of chitin synthesis, neurotoxicity via octopaminergic pathways, midgut epithelial disruption leading to larval mortality, and inhibition of detoxification enzymes, such as glutathione S-transferases (GSTs) and esterases (Ranganathan et al. 2022; Chen et al. 2025). Extracts from *Laurencia* sp. and *Prasiola crispa* have shown potent larvicidal activity, while cyanobacteria produce cyclic peptides with evidenced toxicity to mosquito larvae (Verma et al. 2024).

*Sargassum vulgare* and *Arthrospira platensis* contained phenols, notably quercetin, kaempferol, and resorcinol, tannins, and alkaloid compounds strongly associated with insecticidal activity (Asimakis et al. 2022). Similarly, ethanolic extracts of *Caulerpa sertularioides*, *Sargassum horridum*, and *Laurencia johnstonii* exhibited both insecticidal and repellent effects against the Asian citrus psyllid *Diaphorina citri* (Lu et al. 2021; Poveda and Díez-Méndez 2023). The efficacy of algal biopesticides is often species- and stage-specific. In *Ulva lactuca*, acetone extracts were particularly potent against *Culex pipiens* larvae, while ethanolic and chloroform extracts of the same alga suppressed larval development of *Spodoptera littoralis*, and methanolic fractions inhibited pupation and adult emergence (Costa et al. 2019; Jönsson et al. 2020). Broad-spectrum effectiveness has also been reported against major agricultural pests of the genera *Dysdercus* and *Spodoptera*, where extracts of *Padina pavonica*, *Caulerpa scalpelliformis*, *Sargassum tenerrimum*, *Ulva fasciata*, and *U. lactuca* induced nymphal mortality, adult mortality, and reduced fecundity, hatchability, and lifespan (Ansari et al. 2012; Sarwar 2015; Ramkissoon et al. 2017). Remarkably, Sharanappa et al. (2024) investigated the insecticidal activity of cyanobacterial extracts against *Spodoptera frugiperda*. The petroleum ether extract of *Nostoc muscorum* had the lowest LC_50_ value of 155.22 ppm.

Additionally, microalgae and cyanobacteria have demonstrated valuable arthropod management potential. Extracts of *Arthrospira* sp., *Nostoc* sp., and other genera possess proteinaceous and phenolic compounds known to disrupt insect metabolism, feeding behavior, and hormonal regulation (Esserti et al. 2017; Ibraheem et al. 2017; Sharanappa et al. 2024). In some cases, algal extracts display activity comparable to synthetic insecticides, offering viable replacements within IPM systems (Ahmadi et al. 2015; Ngala et al. 2016). Long-term interest in the insecticidal properties of algae is well documented, with early studies identifying toxic effects of algal metabolites on stored-product insects and agricultural pests (Morgan and Tarjan 1980; Featonby-Smith and Van Staden 1983; Radwan et al. 2011, 2012; Baloch et al. 2013). Collectively, these findings emphasize that algal insecticidal and acaricidal biopesticides offer versatile, multi-target, and environmentally compatible tools for sustainable pest management. The chemical diversity, low ecological footprint, and demonstrated effectiveness across stages of multiple pest species support the integration of algae-derived insecticides into modern agricultural IPM frameworks.

### Herbicidal activity

**Fig. 4:**
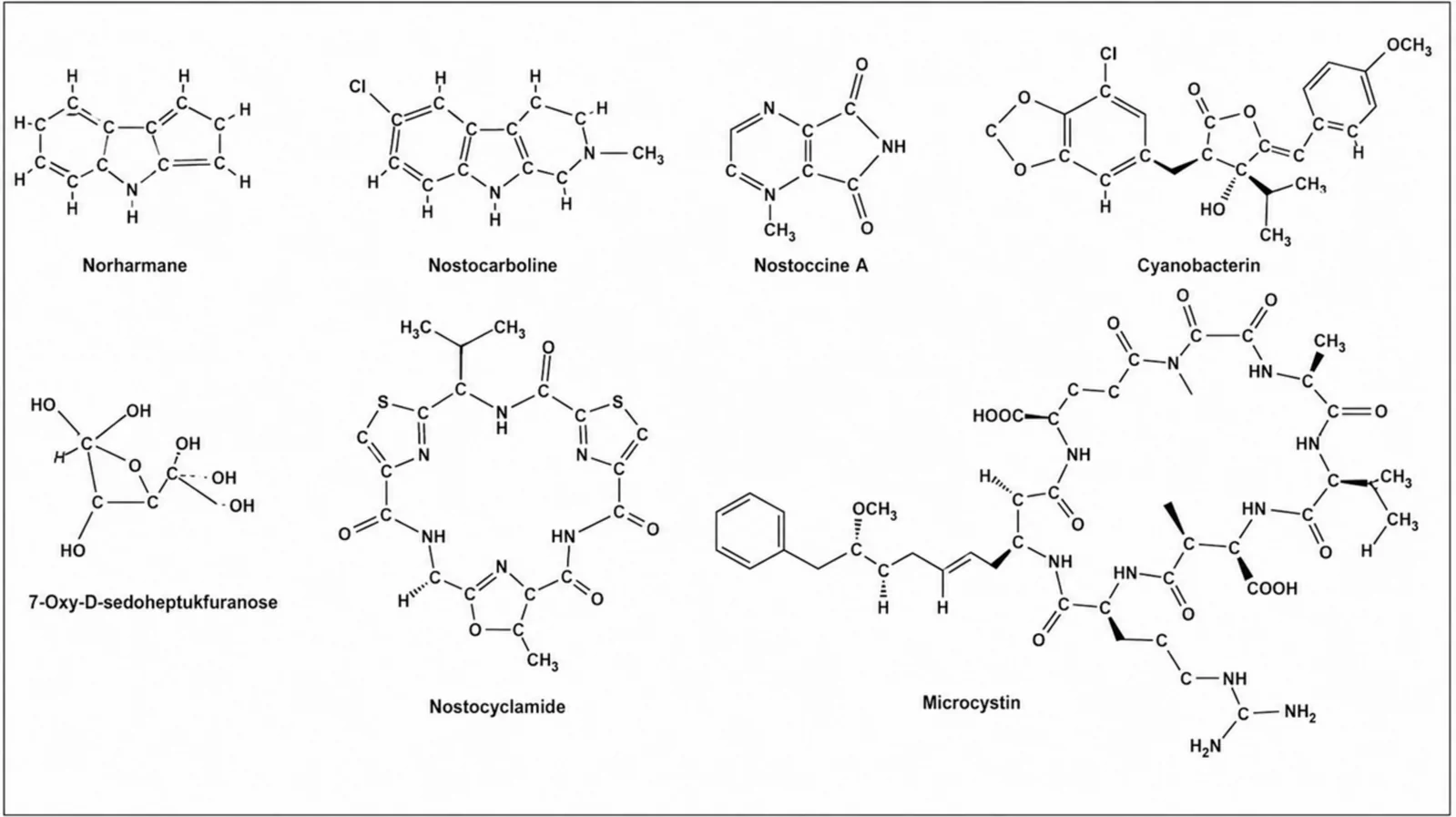
Chemical structures of key cyanobacterial secondary metabolites relevant to biopesticidal activity. Depicted are representative bioactive compounds produced by cyanobacteria, including the alkaloids norharmane, nostocarboline, and nostocine A; the chlorinated aromatic metabolite cyanobacterin; the rare sugar 7-deoxy-d-sedoheptulofuranose; and the cyclic peptides nostocyclamide and microcystin. These metabolites illustrate the structural diversity of cyanobacterial natural products associated with antimicrobial, herbicidal, and cytotoxic modes of action in biopesticide application. These structures highlight functional features relevant to pesticidal activity, including alkaloid scaffolds associated with phytotoxicity, chlorinated aromatic groups linked to photosynthetic inhibition, rare-sugar structures targeting primary metabolism, and cyclic peptides associated with cytotoxic or enzyme-inhibitory effects

Natural bioherbicides have emerged as an important tool in the integrated management of herbaceous weeds, which represent a major agricultural challenge due to their ability to suppress crop growth and significantly reduce yields (Dayan et al. 2009). In this regard, microalgae and cyanobacteria provide a rich source of allelochemicals and cyanotoxins with potent herbicidal properties. Algal metabolites (e.g., cyanotoxins) that exhibit cytotoxicity are promising candidates for herbicidal activity (Singh et al. 2017). Additionally, other cyanobacterial antimetabolites, such as 7-deoxysedoheptulose (7dSh), have demonstrated herbicidal potential by inhibiting the shikimate pathway enzyme dehydroquinate synthase, which is crucial for aromatic amino acid biosynthesis in plants (Casanova et al. 2023). Many cyanotoxins function ecologically as allelochemicals, inhibiting competing macrophytes, algae, and microbes in aquatic ecosystems (Berry et al. 2008; Rastogi and Sinha 2009). Several of these compounds have demonstrated strong phytotoxicity toward terrestrial weeds. For example, the indole alkaloid norharmane (Fig. 4), isolated from *Synechocystis aquatilis*, inhibits the cyanobacteria *Microcystis aeruginosa* and *Oscillatoria limnetica*, as well as the green algae *Chlorella vulgaris* and *Ulothrix* sp., and suppresses germination and growth in terrestrial weeds such as *Avena fatua*, *Portulaca oleracea*, *Echinochloa crus-galli*, *Plantago lanceolata*, and *Amaranthus retroflexus*, while disrupting metabolic processes in *Arabidopsis thaliana* (Casanova et al. 2023).

Other unique metabolites, such as the rare sugar 7dSh from *Synechococcus elongatus*, impair the growth of cyanobacteria, yeasts, and land plants, illustrating the broad-spectrum phytotoxicity potential of microalgal allelochemicals (Brilisauer et al. 2019). Moreover, methanolic extracts of *Nostoc* ATCC 53789 inhibited the development of mixed grass seedlings by reducing culm height and root growth without affecting initial seed germination (Biondi et al. 2004), demonstrating extract-level herbicidal potential. However, despite these promising activities, a notable limitation of cyanobacterial toxins is their potential toxicity to non-target organisms, including animals, humans, and crops, as well as their capacity for bioaccumulation within food chains (Berry et al. 2008; Asimakis et al. 2022; Tiwari et al. 2022). Therefore, while microalgal allelochemicals, including PSII inhibitors, hormone mimics, mitotic inhibitors, and primary metabolism disruptors, represent a promising and chemically diverse source of next-generation bioherbicides, their development must carefully balance efficacy with environmental and toxicological safety considerations.

Across the reviewed studies, the strongest translational evidence is currently available for algal extracts tested against phytopathogenic fungi, plant-parasitic nematodes, and selected bacterial diseases under greenhouse or plant-based assays. In contrast, many antibacterial and antiviral reports remain at the in vitro screening stage. Dose–response data are inconsistently reported, limiting direct ranking of compound classes. Future studies should prioritize standardized LC₅₀, EC₅₀, MIC, and disease-reduction metrics under greenhouse and field conditions.

A comparative analysis of the reported potency values (Table 7) reveals substantial variation in biological activity across algal compound classes and target organisms. Polyunsaturated fatty acids and phenolic compounds frequently demonstrate strong antibacterial and antifungal activity, often with MIC values in the low µg/ml to mg/ml range under in vitro conditions. Sulfated polysaccharides, including laminarin, fucoidan, and carrageenans, primarily exhibit antiviral and plant immune-eliciting effects rather than direct toxicity. In contrast, alkaloids, brominated diterpenes, and terpenoid-rich extracts often display stronger nematocidal, insecticidal, or herbicidal activity, with measurable LC₅₀ or EC₅₀ values in controlled assays. However, direct ranking remains challenging because extraction procedures, assay systems, target developmental stages, and exposure conditions vary considerably among studies.

**Table 7:** Comparative biological potency of algal-derived bioactive compounds across target pests and pathogens

Compound class	Representative compound/extract	Algal source	Target organism	Target type	Assay	Potency metric	Reported activity	Reference
Polyunsaturated fatty acids	EPA/DHA extract	*Isochrysis galbana*	*Listeria monocytogenes*	Model bacteria	Broth dilution	MIC	100–1000 µg/ml	Alsenani et al. (2020)
Carotenoid	β-carotene	*Dunaliella salina*	*Pseudomonas syringae*	Plant pathogen	In vivo	Disease reduction	65.7% incidence reduction	Ambrico et al. (2020)
Sulfated glycolipid	Sulphoglycerolipid	*Sargassum wightii*	*Xanthomonas oryzae*	Plant pathogen	Leaf assay	EC₅₀/inhibition	N/A	Arunkumar et al. (2005)
Sulfated polysaccharide	Laminarin	*Laminaria digitata*	*Botrytis cinerea*	Plant fungus	Greenhouse	Disease suppression	N/A	Paulert et al. (2007)
Phenolics/phlorotannins	Crude extract	Brown algae	*Fusarium oxysporum*	Plant fungus	Growth assay	EC_₅₀_	N/A	Shah et al. (2021)
Brominated terpenes	Sequiterpenes/ethanolic extracts and fractions	*Laurencia johnstonii*	*Aedes aegypti* larvae	Insect	Larvicidal assay	LC_50_	240 µg/mL	Gonzalez-Castro et al. (2024)
Brominated diterpenes	Diterpene fraction	*Jania rubens*	*Meloidogyne javanica*	Nematode	Mortality assay	LC₅₀	N/A	Awad (2004)
Alkaloids	Norharmane	Cyanobacteria	*Amaranthus retroflexus*	weed	Herbicidal assay	EC₅₀	Not available	Casanova et al. (2023)
Terpenoid/polyphenol extract	Crude extract	*Laurencia* spp.	*Aedes aegypti*	Insect	Larvicidal assay	LC₅₀	Not reported	Verma et al. (2024)
Halogenated terpenes	(+)-obtusol	*Laurencia dendroidea*	*Aedes aegypti* larvae	Insect	Larvicidal assay	LC_50_	3.5 ppm	Salvador-Neto et al. (2016)

## Formulation of algal biopesticides

**Fig. 5:**
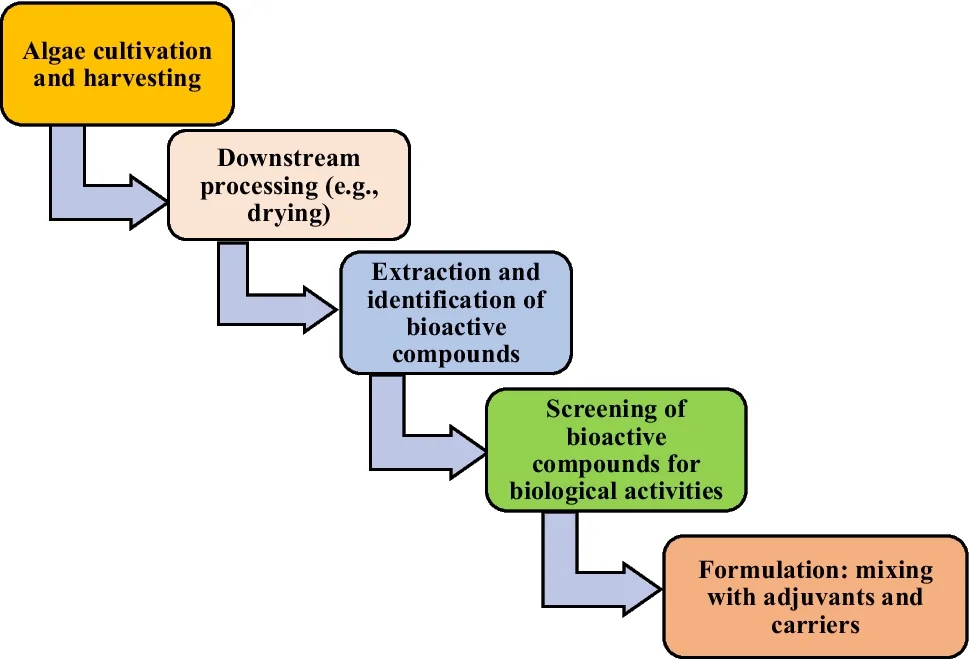
Schematic illustration outlining the sequential steps to produce algae-based biopesticides, from algal cultivation and biomass harvesting to bioactive compound extraction, formulation, and final product development

Macroalgae or microalgae cultivation occurs in a liquid medium (e.g., water) in the presence of adequate nutrients and optimized environmental factors for high biomass production. After harvesting from the growth medium, the biomass undergoes further downstream processing (e.g., drying). The biomass is a rich source of biochemically active compounds (such as terpenoids and polyphenols), which can be recovered using appropriate extraction techniques (e.g., aqueous, solvent, or microwave-assisted extraction) to produce biopesticides with varying biological activities (Sekar et al. 2025). In the formulation of biopesticides, the biocontrol agents should be economical, shelf-stable, user-friendly, target-specific, and effective in the destruction of desired pests (Mishra et al. 2020). A biopesticide is prepared by mixing an algal extract component of a known biological activity with a carrier and adjuvants (e.g., stabilizers and binders) to ensure the survival of the biological agent and improve the lifespan and efficiency of the finished product (Mishra et al. 2020) (Fig. 5). Biopesticides exist in two major types, namely dry and liquid-based formulations (Knowles 2008). Generally, biopesticides exist as powders, emulsions, hydrogels, aqueous suspensions, capsules, or nano-capsules (Hernandez-Tenorio et al. 2022). Numerous techniques, such as direct application, dilution in water, and seed dressing formulations, are employed to deliver biopesticides to crops, depending on the type of biocontrol agents.

## Techno-economic feasibility of algae-based biopesticides

TEA is a critical tool used to assess the technical feasibility and commercial viability of any industrial bioprocess. A standard TEA evaluates both the economic performance and the cost–benefit profile of a process, enabling informed decision-making for scale-up and commercialization. A typical TEA framework begins by defining system boundaries, which generally encompass both upstream and downstream processing steps. The input assessment covers two major cost components, namely capital expenditure (CapEx) and operational expenditure (OpEx). The CapEx includes initial investments such as cultivation systems (medium, reactors, and infrastructure), harvesting technologies, extraction units, bioproduct development facilities, electricity requirements, and solvent usage, whereas the OpEx involves the recurring costs associated with running the process, including labor, maintenance, utilities, and consumables.

The output of a TEA usually provides estimates of product cost, net profit, payback period, interest, and potential carbon-credit gains. These indicators collectively determine whether the process is commercially viable. In many bioprocesses, direct profitability may not be achieved in the first cycle; however, consecutive production cycles often stabilize costs and may yield profit. Additionally, indirect economic benefits such as carbon credits or tax incentives can further improve feasibility (Vázquez-Romero et al. 2022). Within the algal biorefinery field, numerous TEA studies have been reported for biofuel production, bioplastics, CO₂ sequestration, wastewater treatment, and pigment extraction (Bhatt et al. 2022; Figueroa-Torres and Theodoropoulos 2023). However, TEA studies focused specifically on biopesticide production from microalgal biomass remain scarce, with only a few preliminary assessments available.

In this section, we critically examine the limited TEA studies on algae-based biopesticide production, highlight existing gaps, and emphasize the need for comprehensive economic evaluations to support future commercialization. Further, Duarte et al. (2021) evaluated the potential of *Asparagopsis armata* as a biopesticide for weed control and conducted an economic analysis. The outcomes showed significant biopesticide efficiency against *Thellungiella halophila* and impaired photosynthesis, carotenoid metabolism, and induced oxidative stress. They also suggested that using this approach can be aligned with the circular bioeconomy framework. However, TEA studies for specific biopesticides are lacking, and only a few experimental reports exist for biofertilizers. For example, Romero-Garcia et al. (2022) conducted a TEA using algal biomass to produce biofertilizer. In their study, an urban microalgae-based wastewater treatment plant using *Scenedesmus almeriensis* biomass produced 2000 t year⁻^1^ of sludge containing 20% biomass and 80% sludge, which is then processed through a continuous biorefinery modeled in ASPEN Plus V9. The simulated process includes biomass storage, high-pressure homogenization pretreatment (200 bar), enzymatic hydrolysis using Alcalase 2.5 L and Flavourzyme 1000 L at 50 °C, pH control using Ca(OH)₂ and H₂SO₄, solar-thermal-based heating, centrifugation for separation, and final storage and packaging. Material and energy balances indicate annual consumption of 37.91 t of enzymes, 22.08 t of Ca(OH)₂, 6.97 t of H₂SO₄, and 8.19 × 10⁷ kJ of thermal energy, producing 1645.95 t year⁻^1^ of biofertilizer and 483.51 t year⁻^1^ of solid by-product. Equipment costs (APEA-generated) total €1,088,448.80, including major units such as homogenizers, centrifuges, reactors, and storage tanks, along with an additional €80,000 for the packaging machine and seven solar-thermal collectors (€650 each). Total CapEx reaches €9,648,523.33, dominated by design, engineering, and procurement (33.25%), electrical installations (13.49%), contingencies (15.44%), and instrumentation (10.73%). OpEx includes labor (operators at €23.41 h⁻^1^, supervisors at €26.45 h⁻^1^), utilities (electricity at €0.12 kWh⁻^1^, water at €EUR m^3^), and consumables (Ca(OH)₂ at €65 t⁻^1^, H₂SO₄ at €73 t⁻^1^), with biomass sludge and enzymes treated as variable-cost factors for sensitivity analysis. Economic indicators such as net present value (NPV), internal rate of return (IRR), payback value (PPV), and profitability index (PI) were evaluated by varying biomass cost (€0.2–1 kg⁻^1^ sludge), enzyme cost (€10–25 kg⁻^1^), and sale price of the amino-acid-rich biofertilizer (€2.5–7.5 kg⁻^1^). Profitability requires a minimum sale price > €3.5 kg⁻^1^ to achieve NPV > 0 and IRR > 0, while attractive investment performance (PPV 6–7 years, IRR > 15%) demands a selling price of €3.9–4.4 kg⁻^1^. Overall, their TEA demonstrates that large-scale amino acid biofertilizer production from microalgal sludge is technically viable but economically sensitive to biomass and enzyme costs, with profitability strongly dependent on product pricing and operational efficiency. Notably, previous studies on macroalgae-based biopesticides largely remain at laboratory scale, without TEA assessments, representing a major knowledge gap, whereas Arias et al. (2024) conducted TEA analyses specifically for macroalgal biostimulants. They performed a comprehensive evaluation of macroalgal (seaweed) valorization for biostimulant production, using process modeling, TEA, and life cycle assessment (LCA), demonstrating both economic feasibility and environmental benefits. The study showed that macroalgal biostimulants are commercially viable, with a minimum selling price comparable to existing products (HALDEL®, Agroplasma®, DEVELOP®), ranging from $2.45/L to $2.61/L. It was also noted that the use of conventional media for algal cultivation and biopesticide production is not economically viable and is difficult to compete with conventional pesticides. Therefore, it is necessary to design an economically viable approach to produce low-cost biopesticides using an algal source (Arias et al. 2024).

### Advanced techno-economic analysis approach for cost-effective algal biopesticide production

The cost of biopesticide production can be reduced by the integration of wastewater and the design of multiple biorefinery approaches. For example, the production of biofuel with the addition of biopesticides can be a more economical approach compared to a single biorefinery system. Recent literature also suggested similar thoughts. For example, Mohanty and Mohanty (2023b) proposed the dual strategy to produce biofuels and biopesticides using *Chlorella thermophila*. The microalga was initially cultivated in dairy wastewater, and the harvested biomass was used for biopesticide production for the control of bacterial infection (rice blight) in rice plants. The remaining biomass was utilized for biocrude oil production using hydrothermal liquefaction. The authors also proposed a biorefinery model for commercial possibilities. The results showed that the produced biopesticides from algal biomass reduce the growth of *Pantoea agglomerans*, *Xanthomonas oryzae*, and two phytopathogens. Moreover, a 28% biocrude oil yield was reported. The economic assessment of this approach suggests possible implementation on a commercial scale.

As mentioned, very few studies have been carried out on the TEA of biofertilizer production using algal biomass. Regarding the same, recently, a microalgae-triple superphosphate (TSP) biofertilizer production plant was conducted by Junior et al. (2025) using the percentage of delivered equipment cost method, integrating equipment-level estimates from Aspen Plus 14.1 with financial indicators calculated in Excel® to establish the minimum selling price (MSP) required to achieve a payback period of 3.6 years. The economic evaluation incorporated a 10% annual discount rate, and annual operating expenses derived from raw material consumption was 4153 million kg/y of microalgal biomass (USD 0.65/kg), 41,184 million kg/y of triple superphosphate (USD 0.75/kg) along with labor (three shifts, USD 8.21/h), electricity demand of 4,380,090 kWh/y (USD 0.084/kWh), and medium-pressure steam consumption of 86,818 × 10^3^ kg/y (USD 6/1000 kg). Equipment procurement totaled USD 1.37 million, while fixed CapEx reached USD 6450 million, with total CapEx amounting to USD 7417 million after accounting for working capital. Outputs of the integrated process included 51.45 million kg/y of granulated biofertilizer and 0.0117 million kg/y of carbon credits, the latter valued at USD 0.265/kg. Under these conditions, the MSP of the biofertilizer was calculated at USD 1.06/kg, approximately, which was 41% higher than commercial TSP. Depreciation-excluded annual product cost was USD 39,891 million, return on investment was 19.8% per year, NPV was USD 0.75 × 10⁶, and the payback period met the 3.6-year criterion. Sensitivity analysis revealed TSP cost as the dominant economic driver, responsible for 89% of annual operating costs and causing ± 20.4% variation in MSP when fluctuated by ± 25%, whereas all other variables collectively influenced MSP by 0.2–3%. Overall, their TEA outcomes concluded that integrating wastewater-derived microalgal biomass with TSP yields a technically feasible and economically attractive biofertilizer pathway, especially when carbon credits, waste valorization benefits, and improved nutrient-use efficiency are considered, positioning the process as a competitive and sustainable alternative to conventional phosphate fertilizers (Junior et al. 2025).

## Comparative strategies for reducing biopesticide production costs: a conceptual economic assessment

(i) Scenario I: Conventional algal biorefinery approach for biopesticide production

Conventional microalgal cultivation typically requires defined salts and synthetic media to support growth. Microalgae can grow under phototrophic, heterotrophic, and mixotrophic modes; however, literature consistently shows that mixotrophic cultivation yields higher biomass in species such as *Chlorella* (Sharma et al. 2016), *Tetraselmis indica* (Goswami et al. 2023), *Dunaliella* sp. (Gonabadi et al. 2022), and *Picochlorum* sp. (Goswami et al. 2022). Despite this advantage, supplying external organic carbon significantly increases operational costs.

**Fig. 6:**
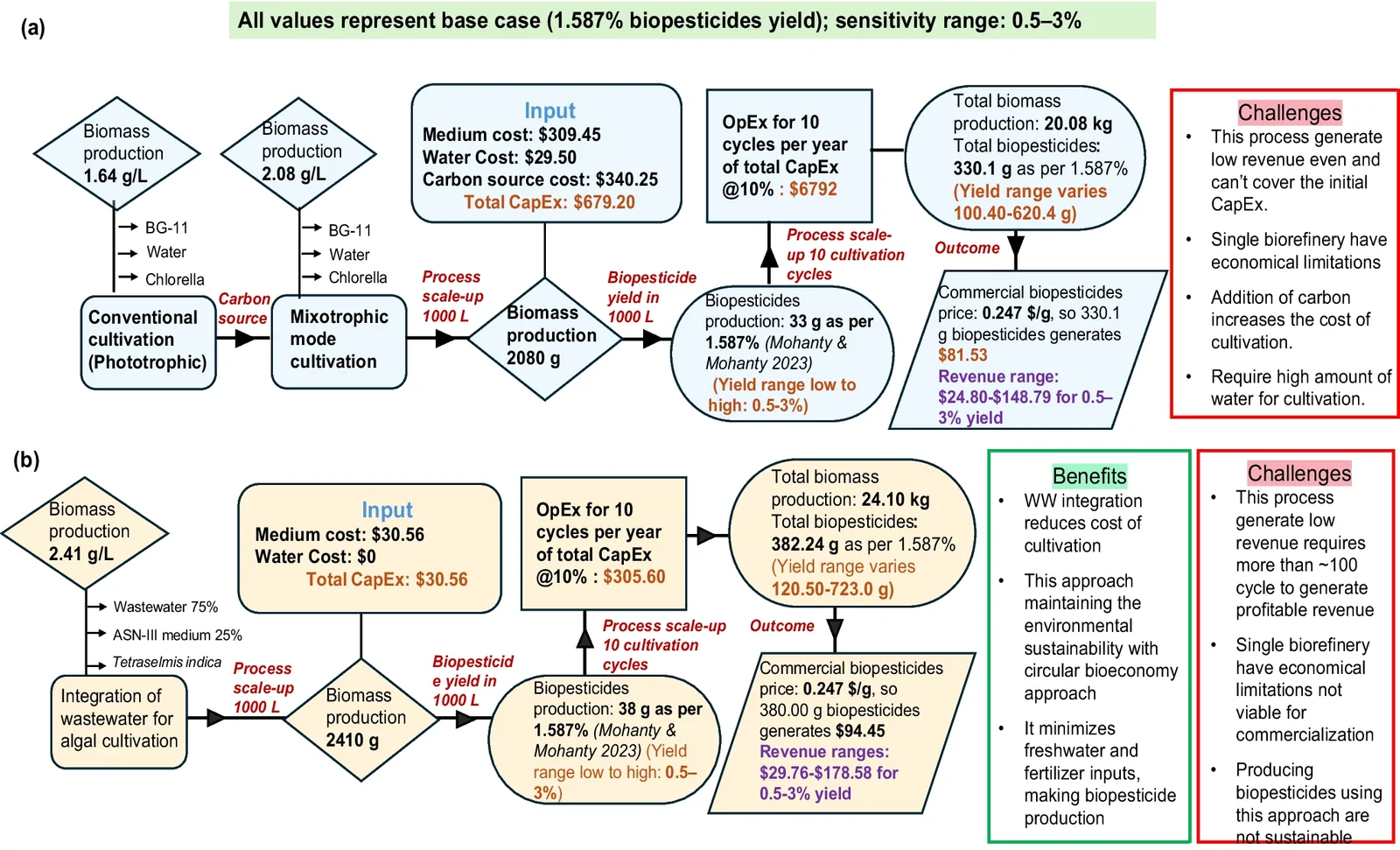

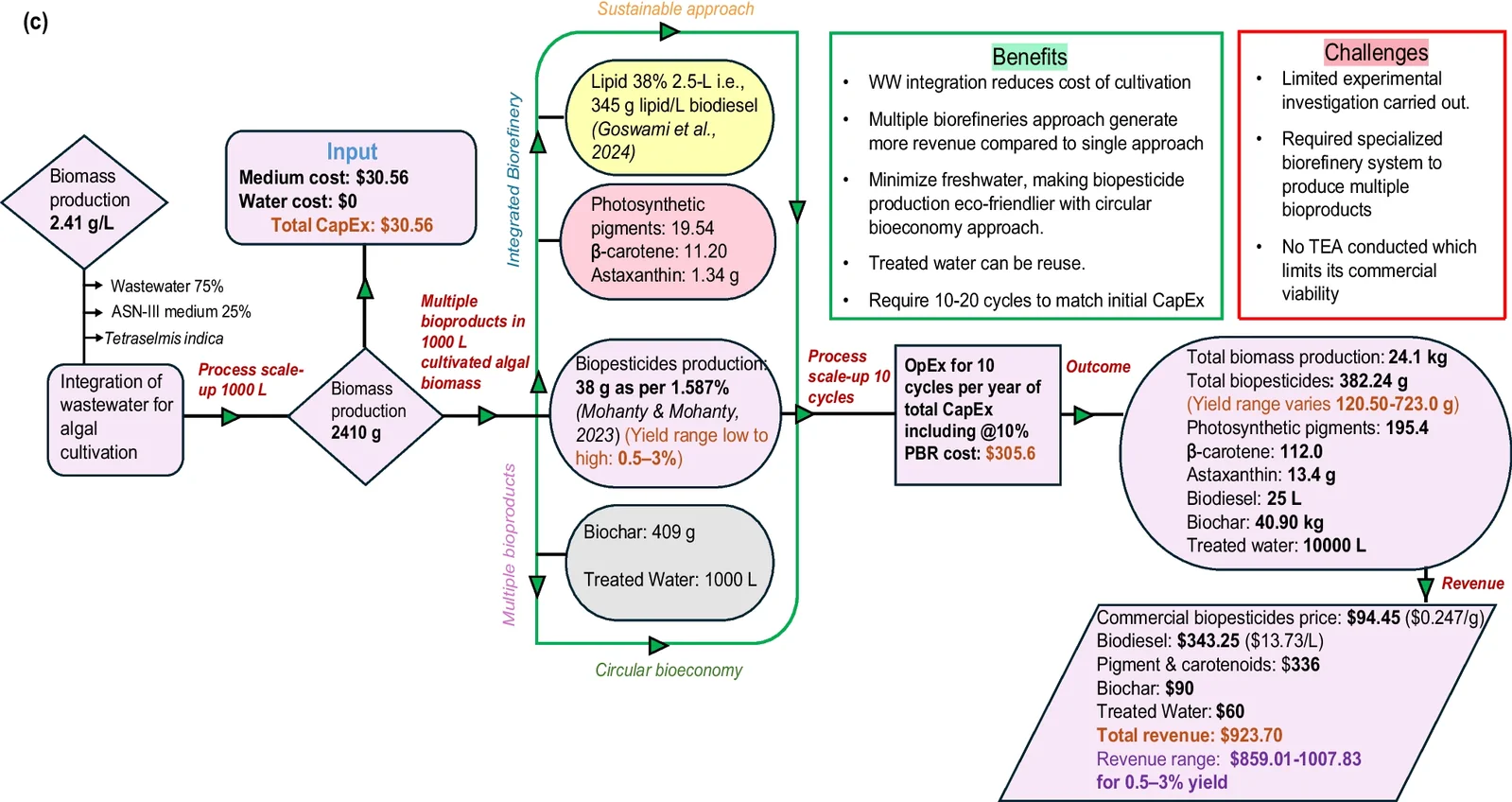
Schematic diagrams illustrating different algal biorefinery approaches for biopesticide production. The base-case biopesticide yield (1.587% w/w, adapted from Mohanty and Mohanty 2023) is used for calculations, while a sensitivity analysis (0.5–3% yield) is incorporated to account for variability in conversion efficiency

Based on earlier literature, market price, and conceptual calculations, we designed a cost assessment for algae-based biopesticide production scaled to a 1000 L system and subsequently to 10 cultivation cycles (Fig. 6a). In scenario I, conventional medium was supplemented with wastewater, and the medium cost was calculated using the chemical requirements for 1000 L based on Sigma pricing. The total CapEx for a single 1000 L unit was $679.20, producing 2.08 g/L biomass (2080 g per batch) under 0.5 g/L carbon supplementation (Sharma et al. 2016). Scaling to 10 cycles resulted in 20.08 kg biomass with an OpEx of $6792. Using the reported yield of 1.587% biopesticides from algal biomass (Mohanty and Mohanty 2023b), scenario I would generate 330.1 g of biopesticides, equivalent to $81.53 in revenue (https://www.vegetables.cornell.edu/pest-management/disease factsheets/biopesticides/biopesticide-application-costs/). It is important to note that this yield represents a single literature-derived value and may vary depending on species, cultivation conditions, and extraction efficiency. To account for this uncertainty, a conceptual sensitivity range was considered, including a low case (0.5%), base case (1.587%), and high case (3%) biopesticide yield. Quantitatively, this corresponds to a biopesticide production range of 100.4–602.4 g and a revenue range of $24.80–148.79, which remains insufficient to offset the high operational expenditure ($6792), thereby reinforcing the economic infeasibility of scenario I across all yield conditions.

However, it is important to acknowledge that systematic studies quantifying the influence of microalgal species selection, extraction methodologies, and target bioactive compounds on biopesticide yield are currently very limited in the literature. As a result, the adopted sensitivity range represents a conceptual approximation rather than a fully validated parameter space, and actual yields may vary significantly under different process conditions. However, it is widely recognized that both species selection and extraction methodology can significantly influence the recovery of bioactive compounds, including biopesticides. The major limitations of microalgae-based products are primarily linked to high biomass production costs, energy-intensive harvesting, and expensive downstream processing. For example, autotrophic cultivation often results in relatively low biomass productivity, while biomass recovery and metabolite extraction are highly energy- and cost-intensive processes. In addition, conventional extraction techniques rely on large volumes of organic solvents, raising both economic and environmental concerns. These combined factors significantly increase the overall production cost of microalgae-derived biopesticides, often making them less competitive than conventional chemical products and limiting large-scale agricultural application (Gonçalves 2021).

Comparison with commercially available natural biopesticide products (Table 8), price ranges between 2.4 and 247 USD/kg or L (Cornell CALS (College of Agriculture and Life Sciences n.d.; https://www.vegetables.cornell.edu/pest-management/disease-factsheets/biopesticides/biopesticide-application-costs/) further indicates that, despite high market pricing, the low biomass-to-product conversion efficiency in scenario I significantly limits revenue generation, making large-scale commercialization economically unattractive. Overall, scenario I is neither economically nor environmentally viable and fails to recover even the initial CapEx or the cumulative OpEx across 10 cycles.

**Table 8:** Market prices and normalized cost structure of commercially available biopesticide products used for techno-economic benchmarking

Commercial biopesticides products	Original price	Original unit	Normalized quantity	Price per unit (USD/kg or USD/L)	Reference		
Actinovate AG	$126.00	18 oz	0.51 kg	$247/kg	Original product names, prices, and unit sizes were compiled and adapted from Cornell CALS (College of Agriculture and Life Sciences) n.d.) Biopesticide Application Costs database (https://www.vegetables.cornell.edu/pest-management/disease-factsheets/biopesticides/biopesticide-application-costs/)		
AgriPhage	$34.90	1 qt	0.946 L	$36.9/L			
AgriPhage-CMM	$317.50	2.5 gal	9.46 L	$33.5/L			
Carb-O-Nator	$58	5 lb	2.27 kg	$25.6/kg			
Bio-Tam 2	$195	5 lb	2.27 kg	$86/kg			
Double Nickel 55 WDG	$39.00	1 lb	0.4536 kg	$86/kg			
Howler	$60.00	5 lb	2.27 kg	$26.0/kg			
Kaligreen	$58.95	5 lb	2.27 kg	$26.0/kg			
Mildew Cure	$20.95	1 qt	0.946 L	$22.1/L			
Organocide	$15.99	32 oz	0.946 L	$16.9/L			
RootShield Granules	$370.00	40 lb	18.14 kg	$20.4/kg			
SoilGard	$79.00	5 lb	2.27 kg	$34.8/kg			
TerraNeem EC	$52.00	1 gal	3.785 L	$13.7/L			
Cueva copper	$114.00	2.5 gal	9.46 L	$12.1/L			
JMS Stylet Oil	$49.00	2.5 gal	9.46 L	$5.18/L			
Kocide 3000-O	$108.00	10 lb	4.54 kg	$23.8/kg			
Nordox 75 WG	$169.00	20 lb	9.07 kg	$18.6/kg			
Microthiol Disperss	$33.00	30 lb	13.61 kg	$2.43/kg			
Product system/scenario	Biomass yield after 10 cultivation cycles (kg)	Biopesticide yield (%) Lower: Calculated: Higher range	Biopesticide produced (kg)	Price basis (USD/kg)	Revenue (USD)	Key assumption	Reference
Scenario-I (Conventional algal biopesticides)	20.08	0.5**/**1.587**/**3	0.1004**/**0.331**/**0.602	24.80**/**81.53**/**148.79	Same as biopesticide	Single product	
Scenario II (Wastewater-based cultivation)	24.10	0.5**/**1.587**/**3	0.1205**/**0.382**/**0.7230	29.76**/**94.45**/**178.58	Same as biopesticide	Wastewater-based cultivation	
Scenario III (Multi-product biorefinery)	24.10	0.5**/**1.587**/**3	0.1205**/**0.382**/**0.7230	29.76**/**94.45**/**178.58	859.01**/**923.70**/**1007.83	Multi-product biorefinery	

(ii) Scenario II: Integrating algal biorefinery with wastewater-based cultivation

Scenario II incorporates a more sustainable and cost-effective strategy by cultivating *Tetraselmis indica* using a 75% wastewater and 25% ASN-III medium blend (Goswami et al. 2024). This approach achieved a biomass productivity of 2.41 g/L, equating to 2410 g per 1000 L (Fig. 6b). The CapEx for medium preparation in this scenario was drastically reduced to $30.56 per 1000 L using wastewater and reflecting minimal dependence on commercial nutrients. Across 10 cultivation cycles, the OpEx was $305.60, producing 24.10 kg biomass. Using the same 1.587% conversion factor (Mohanty and Mohanty 2023b), scenario II yielded 382.24 g of biopesticides, generating $94.45 in revenue. Considering the same sensitivity range (0.5–3%), the projected biopesticide output and revenue would vary proportionally, indicating that although wastewater integration reduces production costs, economic viability remains strongly dependent on achievable product yields. Quantitatively, this corresponds to a biopesticide production range of 120.5–723.0 g and a revenue range of $29.76–178.58, despite reduced operational costs. However, when comparing with other available commercial biopesticide products (Table 8), scenario II still operates at the lower end of revenue performance, highlighting that cost reduction alone is insufficient to achieve industrial competitiveness without improving product diversification or conversion efficiency. Although significantly more economical than scenario I, scenario II still requires approximately 100 cycles to recover CapEx + OpEx. This assessment highlights that while wastewater integration reduces costs, producing only a single value-added product (biopesticides) remains insufficient for commercial viability.

(iii) Scenario III: Multi-product algal biorefinery integrated with wastewater systems

Scenario III builds upon scenario II but adopts a multi-product biorefinery approach, utilizing the same cultivation conditions, biomass output, CapEx, and OpEx (Fig. 6c). As demonstrated by Goswami et al. (2024), *Tetraselmis indica* contains several additional high-value biomolecules, including pigments and antioxidants, enabling multiple revenue streams in addition to biopesticides (382.24 g). In this conceptual multi-product assessment, the combined value of extracted products generates $923.70 across 10 cycles. Even when accounting for variability in biopesticide yield (0.5–3%), the inclusion of multiple co-products significantly buffers economic risk by reducing reliance on a single conversion factor, thereby improving overall process robustness. Under this sensitivity range, the total revenue varies between $859.01 and $1007.83, indicating that scenario III is comparatively less sensitive to yield fluctuations due to the contribution of multiple value-added products. This multi-product approach reduces the system’s overall sensitivity to uncertainties in single-product yields, thereby improving the robustness of TEA projections despite limited experimental validation of individual conversion efficiencies. While this approach still requires approximately 10–20 additional cycles to offset CapEx and OpEx, it substantially improves revenue generation compared to scenarios I and II. Compared to commercial biopesticide benchmarks (Table 8), scenario III approaches the upper revenue performance range due to cumulative valorization rather than increased biopesticide price, demonstrating the economic advantage of integrated biorefinery strategies. Nevertheless, this strategy also demands specialized extraction and separation processes. Importantly, very few studies have experimentally demonstrated such multi-product valorization, and almost no TEA assessments exist for integrated multi-product algal biorefineries, identifying a significant research gap.

Price per unit was calculated as: $\frac{Original\ price\ (USD)}{Normalized\ mass\ (kg)\ or\ volume\ (L)}$  

(iv) Scale-up constraints and process limitations

Despite promising conceptual TEA outcomes, the industrial translation of algal biopesticide production systems faces several critical scale-up challenges. At larger cultivation volumes, factors such as light distribution, temperature control, water availability, and nutrient supply become increasingly difficult to manage. In addition, self-shading effects and mass-transfer limitations significantly reduce photosynthetic efficiency and biomass productivity compared with laboratory-scale systems (Samoraj et al. 2024). For wastewater-based cultivation (scenarios II and III), variability in nutrient composition and the presence of inhibitory compounds can further compromise process stability and reproducibility.

Downstream processing represents a major bottleneck, particularly in multi-product biorefinery systems. Sequential extraction of biopesticides, pigments, and antioxidants increases solvent consumption, energy demand, and separation complexity, thereby impacting overall process economics (Okeke et al. 2022). Moreover, integrated biorefinery designs for biopesticides and other intracellular biomolecules require multistep processing strategies, which add operational complexity and cost.

From an application perspective, microalgae-based biopesticides have been reported to enhance soil fertility and plant productivity by suppressing harmful microorganisms. However, most studies remain limited to laboratory-scale investigations, and there is a lack of comprehensive field-scale validation and long-term agronomic studies. In addition, challenges related to high CapEx and OpEx, technological limitations, regulatory approvals, and limited societal awareness further constrain large-scale adoption (Samoraj et al. 2024).

Overall, the integrated TEA (Table 8 and Fig. 6) indicates that economic feasibility improves progressively from scenario I to scenario III, driven by (i) reduced cultivation costs, (ii) enhanced biomass productivity, and (iii) multi-product valorization. However, scale-up and downstream processing constraints remain key barriers to industrial deployment. Although scenario III approaches commercial benchmark revenue levels, its practical feasibility will depend on overcoming these challenges through optimized cultivation system design, efficient downstream processing, and validated pilot-to-field-scale implementation.

## Regulatory pathways for algal biopesticides

The development and application of algal biopesticides are regulated by intricate legal frameworks established to guarantee their efficacy, safety, and minimal environmental impact (Deeksha et al. 2025). Biopesticides undergo a strict, multi-phase regulatory evaluation before commercialization to guarantee safety for humans, non-target organisms, and the environment. The registration process involves submitting comprehensive dossiers, encompassing product identity, efficacy, and toxicology, to major regulatory authorities, such as the United States Environmental Protection Agency (USEPA) under the Federal Insecticide, Fungicide, and Rodenticide Act (FIFRA) or the European Commission under Regulation EC 1107/2009. Upon review and approval, a certificate is issued, authorizing the product for market release. However, gathering the data requirements for a dossier is a cumbersome process in the development of biopesticides. The specific data requirements that need to be satisfied for biopesticide registration include toxicity and safety data, efficacy data, environmental impact data, and public perception and acceptance data (Kumar 2012; Dar et al. 2021; Ahmed et al. 2022; Butu et al. 2022).

The regulatory agencies conduct rigorous scientific assessments to ensure products meet safety and efficacy standards before entering the market (Arora et al. 2016). Additionally, the agencies have distinct legislation and processes for approving and monitoring biopesticides (Deeksha et al. 2025). The law and policies regulating the use and development of biopesticides differ significantly across countries, and there is no universal regulatory model to streamline their regulation and registration process (Arora et al. 2016). These inconsistencies pose critical challenges to the development pipeline, regulatory approval processes, and large-scale commercialization of algae-based biopesticides in global agriculture (Varghese et al. 2026). Remarkably, in the USA, the EPA requires less data for registration when compared to chemical pesticides (USEPA 2010). Under Regulation (EC) No. 1107/2009, the European Union (EU) biopesticide registration process is considered highly challenging, often perceived as more rigorous than in other jurisdictions. This is largely because biopesticides must fulfill comprehensive data requirements, including assessments of efficacy, environmental impact, and toxicology, like those for conventional chemical pesticides (Hauschild 2012).

## Limitations in the application of algal biopesticides in agriculture

Algal biotechnology provides an efficient means of controlling plant pathogens and pests by producing bioactive compounds through the cultivation of macroalgae and microalgae. Nevertheless, the global acceptance of this technology for commercialization remains a challenge due to some techno-economic and environmental factors, highlighted below.

(i) The cultivation and harvesting of algae require a large amount of water and synthetic nutrients, posing a significant financial burden for large-scale biopesticide production. Therefore, the development of innovative cultivation and harvesting systems is crucial for enhanced biomass and biopesticide productivity at lower cost. Integration of wastewater (medium) can reduce cultivation costs, but the social acceptance of using wastewater-grown algae for bioproduct development still faces a major challenge.
(ii) The extraction of bioactive compounds from algae for the synthesis of biopesticides requires expensive technologies and a large volume of organic solvents, resulting in higher costs of the obtained biopesticides. Consequently, the development of efficient, eco-friendly, and economical techniques for extracting bioactive compounds will pave the way for sustainable and cost-effective biopesticide production.
(iii) Algal extracts may contain metabolites that are hazardous to some crop species. As a result, the safety and effectiveness of algal biopesticides are crucial to avoid the accidental spread of harmful compounds. Therefore, adoption of a metagenomic approach for the selection of suitable target phytopathogens or pests and algal strains with selective antimicrobial or pesticidal activity is imperative, as this technique provides information on the composition and functional activity of phototrophic communities as well as their potential ecosystem threats.
(iv) Algal biopesticides are unstable due to changes in environmental factors, such as humidity and temperature, thereby compromising the effectiveness and stability of the biocontrol agents under field conditions.
(v) The slow action and inconsistent performance of the algal biopesticide discourage farmers and make their field application undesirable when fast pest control is required.
(vi) The high specificity of algal biopesticides to pathogens and pests will allow multiple use of different biopesticides for the concurrent control of a vast array of pests, thus making the process capital-intensive and laborious.
(vii) While algal biopesticides exhibit multi-target bioactivity, challenges persist in scaling, formulation, and field performance. Variability in metabolite profiles due to culture conditions and extraction methods hampers reproducibility. Developing a user-friendly product requires suitable carrier materials to ensure stability. However, finding compatible and low-cost ingredients that do not deactivate the algae is challenging. In addition, the specific bioactive compounds responsible for pesticidal activity are sometimes not fully identified, making it difficult to standardize the product for consistent quality and efficacy. Emerging formulation techniques such as microencapsulation and polymer-based delivery systems improve compound stability. The use of marker compounds, metabolic profiling, and advanced, consistent extraction methods is crucial to ensure effective product standardization and quality.
(viii) The lack of specialized, harmonized guidelines for algae-based products and stringent safety and environmental impact assessments, coupled with high variability in efficacy and complex, expensive registration processes, affects the regulation of algal biopesticides in the agricultural sector. Addressing these challenges requires clearer, tailored regulatory pathways that recognize the unique biological properties of algae while balancing safety with the urgent need for sustainable pest control solutions.
(ix) Algal biopesticides encounter significant storage stability challenges, primarily due to the rapid degradation of bioactive compounds, induced by heat, light, oxygen, and moisture, leading to short shelf-lives and reduced field efficacy. These can be mitigated through advanced formulations and preservation techniques, including encapsulation and coating, the addition of stabilizing agents, lyophilization, spray drying, and wet storage.

## Conclusions and future perspectives

Macroalgae and microalgae are rich sources of a wide range of bioactive compounds (e.g., terpenoids, alkaloids, polysaccharides, phenolics, and peptides) that play key roles in crop protection and productivity. These biochemically active compounds exhibit varying degrees of biological activities, including antibacterial, antiviral, antifungal, nematocidal, and herbicidal activities, through multiple mechanisms. Algal bioactive compounds are eco-friendly, effective, biodegradable, and sustainable biopesticides with a lower risk of pest resistance. Compared with established commercial biopesticides such as *Bacillus thuringiensis* and spinosad, algal-derived biopesticides currently face major barriers, including limited field-efficacy data, sparse regulatory precedent, variable extract composition, and uncertainty regarding scale-up and formulation stability. Therefore, their most realistic short-term role may not be to replace established products directly, but to complement them within IPM. Algal products may be particularly valuable where multifunctionality is advantageous, including simultaneous pathogen suppression, plant immune priming, stress tolerance enhancement, and resistance-management strategies. The following future opportunities can be explored for efficient and sustainable algal biopesticide production.

(i) Further studies should delve into improving the efficacy and stability, as well as easing the regulatory burdens and economic feasibility of algal biopesticides.
(ii) Substantial field trials are imperative to ascertain the efficiency of algal biopesticides on different crops under varying environmental conditions, as well as provide adequate application techniques, formulations, and dosages for maximum benefits of the biocontrol agents.
(iii) Genetic engineering, including clustered regularly interspaced short-palindromic repeats (CRISPR)/Cas nuclease system, can be adopted for improving the production and potency of a particular algal bioactive compound against a specific phytopathogen.
(iv) Nano-formulation of algal bioactive compounds represents a transformative research area for the future of sustainable agriculture, enhancing the stability, solubility, and targeted delivery of natural biopesticides while reducing the required dosage. Algal metabolites can be encapsulated in nano-carriers or green-synthesized as metallic nanoparticles, effectively tackling pests with minimal environmental impacts.
(v) The synergistic effects of a cocktail of different algal extracts could be explored for the development of efficacious and environmentally safe biopesticides.
(vi) Government policies and collaboration among researchers, industry stakeholders, farmers, and academia can expedite the development, adoption, and integration of algal biopesticides into modern agricultural practices.
(vii) Future research should focus on genomic and metabolomic profiling to identify biosynthetic pathways and optimize algal biopesticide production.
(viii) Further TEA and LCA analyses are needed to justify the economic and environmental feasibility of algal biopesticides.

## Data Availability

Not applicable.
